# Comparison of measured and modeled data for carcinogen workplace exposure assessment: a case study on chromium trioxide under REACH and OSH frameworks

**DOI:** 10.1093/annweh/wxag046

**Published:** 2026-06-22

**Authors:** Carolina Zellino, Urs Schlüter, Raffaella Wree, Ann Carolin Dumke, Gudrun Walendzik, Chiara Marelli, Eleonora Pagani, Rocco Loris Del Vecchio, Alessio Carminati, Sabrina Rovelli, Giacomo Fanti, Francesca Borghi, Andrea Cattaneo, Andrea Spinazzè, Domenico Maria Cavallo

**Affiliations:** Department of Science and High Technology, University of Insubria, Via Valleggio 11, 22100 Como, Italy; Federal Institute for Occupational Safety and Health (BAuA), Friedrich-Henkel-Weg 1-25, D-44149 Dortmund, Germany; Federal Institute for Occupational Safety and Health (BAuA), Friedrich-Henkel-Weg 1-25, D-44149 Dortmund, Germany; Federal Institute for Occupational Safety and Health (BAuA), Friedrich-Henkel-Weg 1-25, D-44149 Dortmund, Germany; Federal Institute for Occupational Safety and Health (BAuA), Friedrich-Henkel-Weg 1-25, D-44149 Dortmund, Germany; Team Mastery, Via G. Ferrari 14/B, 22100 Como, Italy; Department of Science and High Technology, University of Insubria, Via Valleggio 11, 22100 Como, Italy; Department of Science and High Technology, University of Insubria, Via Valleggio 11, 22100 Como, Italy; Department of Science and High Technology, University of Insubria, Via Valleggio 11, 22100 Como, Italy; Department of Science and High Technology, University of Insubria, Via Valleggio 11, 22100 Como, Italy; Department of Clinical and Community Sciences – DISCCO - “Clinica Del Lavoro Luigi Devoto”, University of Milan, Via San Barnaba 8, 20122 Milano, Italy; Department of Medical and Surgical Sciences, University of Bologna, Via Pelagio Pelagi 9, 40138 Bologna, Italy; Department of Science and High Technology, University of Insubria, Via Valleggio 11, 22100 Como, Italy; Department of Science and High Technology, University of Insubria, Via Valleggio 11, 22100 Como, Italy; Department of Science and High Technology, University of Insubria, Via Valleggio 11, 22100 Como, Italy

**Keywords:** occupational exposure assessment, Advanced REACH Tool (ART), exposure measurements, exposure modeling, REACH, OSH frameworks, hexavalent chromium

## Abstract

**Introduction:**

Occupational exposure to chromium trioxide (CAS number: 1333-82-0) remains a major concern in the electroplating sector. Risk assessment is regulated under both the REACH Regulation, which focuses on substance-related risks across the supply chain, and Occupational Safety and Health (OSH), which emphasizes workplace-specific exposure. The two regulatory frameworks adopt different approaches, particularly regarding the role of exposure models and monitoring for workplace risk assessment. This study investigated the outcomes of exposure assessment under REACH and OSH, comparing modeled exposure estimates with measured workplace data for occupational exposure to hexavalent chromium, in order to analyze differences in the exposure assessment process and the risk characterization under both approaches.

**Methods:**

The study focuses on four Italian companies involved in metal products manufacturing, which applied to ECHA for authorization to use chromium trioxide. Worker contributing scenarios were defined from Chemical Safety Reports. Exposure estimates were derived using the Advanced REACH Tool, while additionally measured data came from workplace monitoring campaigns, evaluated according to EN 689:2019. Comparisons between exposure estimates and measured data were made following the criteria of level of consistency and level of conservatism. Considerations on risk as risk characterization ratio (RCR) under OSH and excess risk under REACH were also made due to the different regulatory approach.

**Results:**

Model estimates from ART frequently have allow level of consistency with measured workplace concentrations, in some cases by up to two orders of magnitude. RCR values calculated from each exposure measured and estimated data were always <1, although EN 689 testing revealed cases of noncompliance. Excess risk under REACH remained below the tolerable level defined but was closer to it than to the acceptable level.

**Conclusions:**

The analysis highlighted fundamental differences between REACH and OSH: REACH relies mostly on exposure models for registration. For Application for Authorizations, monitoring is more frequently applied. On the other hand, OSH prioritizes monitoring of carcinogenic agents by workplace measurements in any case. Models are useful tools, but their reliability depends amongst others on assessor expertise and contextualization with workplace data. Integrating REACH authorization conditions with OSH monitoring obligations can create a more consistent and effective strategy to protect workers from Cr(VI) exposure. As the concept of risk is framed differently in REACH and OSH, this study highlights how the exposure tools may be used at different levels, leading to mutual benefits for both approaches.

What's Important About This Paper? This study demonstrates that integrating REACH and Occupational Safety and Health (OSH) frameworks creates a superior strategy for protecting workers from exposure to hexavalent chromium, a carcinogen. Focused on four companies with chrome electroplating operations, this study highlights the limitations of exclusive reliance on exposure models, and demonstrates the value of integrating exposure monitoring and modeling for risk evaluation. REACH authorizations strengthen workplace safety by imposing stricter monitoring and control measures than standard OSH requirements.

## Introduction

### Regulatory frameworks for chemicals in Europe

In Europe, chemical risk at workplaces is regulated by the so-called Occupational Safety and Health Legislation (OSH) (referred to Directives 89/391/EC (Council of the European Union 1989); 98/24/EC (Council of the European Union 1998) and 2004/37/EC (European Parliament and Council of the European Union 2004)). OSH requires employers to carry out workplace risk assessments by identifying, evaluating, and controlling—by eliminating if possible or by minimizing—workplace risks, ensuring the safety and health of workers through the implementation of effective risk management. The OSH legislation must be transposed at national level into national regulation, ordinances or laws by Member States who can make additional requirements when they transpose these directives into their national law. In 2007, the European (EU) Chemicals Legislation REACH (Registration, Evaluation, Authorization, Restriction of CHemicals—Regulation (EC) 1907/2006 (European Parliament and Council of the European Union 2006)) entered into force. Different to OSH, REACH is a single, broad-ranging Regulation, that applies directly in each Member State. The main aim of REACH is to ensure a high level of protection of human health and the environment from risks possibly arising from the use of chemicals produced in and imported into the EU, by addressing the responsibilities of all actors in the supply chain, from manufacturers and importers to downstream users and consumers, over the entire life cycle of a substance. Under REACH, manufacturers, importers, and downstream users (ie, mostly employers according to OSH) are supposed to interact with each other, to optimize the protection of workers’ health, enabling risk assessment improvement in OSH as well. As is evident from the brief explanation above, workers' health protection is a common goal of REACH and OSH, and positive interactions and synergies clearly exist between these two regulatory systems. The coexistence of requirements from both frameworks may create parallel obligations for employers, although information generated under one framework may also support duties under the other. However, if OSH requirements are met and documented, in many cases only a review of risk management is needed because of REACH.

### Overall interactions between REACH and OSH from scientific literature

Table 1 reports the main differences and similarities of REACH and OSH frameworks. REACH and OSH interact through a mutual exchange of information regarding exposure scenarios, defined by Article 3.37 of REACH as “the set of conditions, including operational conditions (OCs) and risk management measures (RMMs), that describe how the substance is manufactured or used during its life cycle and how the manufacturer or importer controls, or recommends downstream users to control, exposures of humans and the environment.” These scenarios are central to the chemical safety assessment (CSA) required for registrations of 10 tonnes/yr or more (ECHA 2025a). This synergy was examined during the 5th German REACH Congress in 2021 (Reihlen et al. 2022), focusing on the interlinkages between both frameworks. Participants noted that employer-led risk assessments (eg, workplace monitoring and RMMs) can provide vital data for registrants' CSAs. Conversely, while REACH has improved the quality data (eg, hazard and exposure data) reported in the extended safety data sheets (eSDSs), facilitating OSH risk assessments. To reduce over-interpretation, we acknowledge that the alignment between standardized worker contributing scenario (WCS)/OCs/RMM descriptors and real workplace conditions may be imperfect; this representativeness gap is an inherent limitation of scenario-based modeling and is considered when interpreting model-measurement consistency.

**Table 1:** Differences and similarities of REACH and OSH frameworks.

Feature	REACH regulation	OSH legislation
Legal framework	A single, broad-ranging regulation directly applicable in all EU Member States.	Directives transposed into national law; Member States may add stricter requirements.
Primary aim	Protection of human health and the environment across the entire substance life cycle.	Protection of worker health and safety via workplace risk assessment and management.
Actors	Manufacturers, importers, and downstream users (supply chain focus).	Employers (workplace focus).
Instruments	Chemical Safety Assessment (CSA), extended Safety Data Sheets (eSDS), and Exposure Scenarios (defined by OCs and RMMs). Regulatory tools include Authorization (Annex XIV) and Restriction (Annex XVII).	Workplace Risk Assessment, Workplace Monitoring (environmental and biological), Hierarchy of Control (prioritizing substitution), and Health Surveillance.
Exposure assessment	Predictive and iterative Based on the generation of ESs. It integrates OCs and RMMs for each contributing activity to derive exposure estimates. Tiered modeling: Primarily utilizes standardized use descriptors and mathematical models (Tier 1 or higher) to calculate RCR.	Empirical and site-specific Based on direct workplace monitoring (environmental and biological) and the evaluation of real-time occupational tasks. Direct measurement: Uses actual exposure data collected at the workplace to verify compliance with OELs.
Employer obligation	Often requires a review of risk management to ensure alignment with eSDS.	Mandatory identification and evaluation of all workplace risks.
Interoperability	Facilitates OSH by providing improved hazard and exposure data in the eSDS.	Supports REACH by providing workplace monitoring data for the CSA.

While the CSA provides the technical basis for risk management, regulatory action intensifies for substances of very high concern (SVHCs) via two pathways: authorization under Annex XIV (ECHA 2025b), requiring an Application for Authorization (AfA), or restriction under Annex XVII (ECHA 2025c). For SVHCs, both authorization and restriction under REACH are intended to promote the progressive substitution of these substances with safer alternatives, where such alternatives are available and can be considered suitable from a technical, economic, and risk perspective, or otherwise to ensure that risks are adequately controlled throughout the substance life cycle. When an AfA is granted by the EU Commission following ECHA (European Chemicals Agency) evaluation, use may continue, though typically under improved OCs and stricter RMMs. During the review period, efforts to implement safer alternatives are expected to continue, while OCs and RMMs may need to be further refined or strengthened to ensure adequate risk control. Similarly, the OSH hierarchy of control mandates substitution if feasible, alongside reinforced workplace hygiene, emergency protocols, and specific health monitoring. In this context, the risk reduction potential of REACH and authorization decisions may contribute to the implementation of occupational safety; in support of this, authorization opinions often suggest stricter OCs/RMMs or monitoring obligations. Moreover, authorization opinions suggest stricter OCs/RMMs or monitoring obligations. On this topic, a recent study from Deubner et al. (2024) analyzed 398 AfAs for Cr(VI) substances to investigate the effectiveness of the regulatory tool and its impact on the OSH requirements, examining use conditions, monitoring arrangements, and review reports. The analysis, supported by the ECHA Committees for RAC (Risk Assessment) and SEAC (Socio-Economic Analysis), as well as the EU Commission, underscore the need for significant OSH improvements within the authorization process. Monitoring remains the most frequent ECHA recommendation to verify the effectiveness of OCs and RMMs. If these procedures fail to ensure adequate risk control, REACH restrictions—directly applicable across all Member States—may be introduced to complement OSH risk management provisions and promote harmonize protection. Recent examples include restrictions on diisocyanates and aprotic solvents like NEP (1-ethylpyrrolidin-2-one) and DMAC (N,N-dimethylacetamide) (ECHA 2025c). In general, although OSH establishes nonnegotiable baseline requirements for worker health and safety (Reihlen et al. 2022), a degree of residual risk often persists as not all occupational exposures can be eliminated. The question whether REACH or OSH measures are more appropriate to regulate specific risks for workers is still challenging and needs further evaluation by means of a case to case investigation.

### Approaches for exposure assessment under REACH and OSH

As mentioned above, the exposure assessment process is of paramount importance for meeting the requirements set out under REACH and OSH, and for achieving the common goal of protecting human health and safety. Workplace exposure data may be obtained through both occupational exposure models and environmental or biological monitoring. Although these tools can be applied in both the REACH and OSH contexts, their role within the exposure assessment process differs. In line with ECHA Guidance R.14 (ECHA 2016), exposure assessment under REACH generally follows tiered refinement logic, whereby screening estimates may be refined through more detailed modeling and/or targeted measurements when needed. In this framework, only a limited number of contributing scenarios are evaluated in more detail when Tier 1 modeling indicates that safe use cannot be demonstrated. In such cases, either higher-tier modeling or targeted measurement campaigns are required. Within REACH, exposure models therefore play a central role in estimating exposure in the defined exposure scenarios, while monitoring is favored and often a required condition for authorization. In contrast, under OSH, monitoring represents the cornerstone of exposure compliance control and workplace surveillance. Nonetheless, models can also play a supporting role when measurement data are lacking or insufficient. In this regard, EN 689:2018+AC:2019 (CEN 2019) explicitly acknowledges that an initial exposure characterization may be performed using suitable exposure models to determine whether personal exposure measurements are required. In any case, the reliability of exposure estimates generated by “REACH exposure models” when applied in OSH contexts has been widely debated, and further discussions on their limitations and potential applications have been addressed in previous research (Spinazzè et al. 2017, 2019; Fransman et al. 2022; Koivisto et al 2022; Schlüter et al. 2022, 2023; Koivisto et al. 2025).

The primary aim of this study is to evaluate the reliability of predictive exposure modeling by conducting a direct performance comparison between the Advanced REACH Tool (ART) and on-site monitoring data collected in accordance with the EN 689:2019. Indeed, EN 689 recognizes that exposure estimates may be derived from measurements or from suitable exposure models when sufficient quantitative contextual information is available; in this study, modeling is used as complementary to workplace measurements. While the investigation is contextualized within the differing regulatory requirements of the REACH framework and OSH approach, the analysis focuses on identifying systematic divergences in risk characterization. Specifically, by calculating both the risk characterization ratio (RCR) and excess risk, this research seeks to determine how the choice between modeled and measured data influences risk management decisions and compliance outcomes. Beyond a simplistic “models versus measurements” opposition, this work advocates for a “fit-for-purpose” approach where models serve as essential tools to structure scenarios and prioritize interventions. Establishing the reliability of these estimates is critical: while models offer a systematic framework for assessment, their effectiveness depends heavily on competent handling and precise contextualization with workplace data. In this framework, workplace measurements are used as an empirical benchmark to contextualize model outputs, verify exposure control under real working conditions, and support the interpretation of possible discrepancies between REACH-oriented modeling and OSH-oriented monitoring. Ultimately, bridging the gap between modeled estimates and systematic monitoring- and aligning the requirements of REACH and OSH- provides a more robust, scientifically grounded strategy to ensure the highest level of protection for workers exposed to hazardous substances, such as carcinogens.

## Materials and methods

### Case studies and associated WCS

Four companies located in Northern Italy were selected for the study. These companies were classified based on ECHA use descriptors as “Sector of Use” SU 15 (NACE code C 24, metal products manufacturing, excluding machinery and equipment) and “Product Category” PC 14 (metal surface treatment products). For each company, an AfA for the use of chromium trioxide has been submitted to the ECHA regarding the use of functional chrome plating for technical or decorative purposes. Small and medium-sized companies have been selected for each use. A brief description of the four companies (named A, B, C, and D) is provided below:

- Company A specializes in nickel and chrome electroplating of brass and copper components for thermo-sanitary, medical, and industrial applications. The process employs chromium trioxide to create durable decorative coatings. The company has 23 employees and a plating line of 714 m^2^ within a total area of 2,000 m^2^.
- Company B is a small, family-run business focused on functional chrome plating of various metal substrates (eg, steel, brass, bronze), serving multiple industrial sectors, and also performing mechanical treatments such as sandblasting. Of its 5 employees, 3 work directly on the plating line, which is part of a 1,912 m^2^ facility.
- Company C manufactures aluminium cylinders with hard chrome plating on the barrel, mainly for engines and machinery in the automotive, motorcycle, nautical, and agricultural sectors. 11 employees are involved in the plating activities within a 7,000 m^2^ facility.
- Company D carries out chrome plating of engraved steel press plates used in high-quality decorative laminates. The company employs 65 workers, 6 of whom are directly involved in plating, within a 10,000 m^2^ site (3,000 m^2^ dedicated to plating).

A description of the WCSs and their conditions of use (ie, OCs and RMMs) is reported in Table S1a/b, S2a/b, S3a/b, S4a/b of the Supplementary Materials, section A.

The present case study is intentionally restricted to the processes and WCSs available in the participating companies; therefore, the findings should not be interpreted as representative of all Cr(VI) uses or all plating scenarios.

### Estimated and measured exposure data

Both estimates by modeling and measured exposure data were obtained in this study. Exposure estimates were obtained with ART, while measured data derived from workplace monitoring campaigns conducted under OSH legislation. WCS descriptors and model inputs were derived from the Chemical Safety Report (CSR) and company documentation. However, as full EN689 contextual information was not available for all datasets, deviations from standard reporting requirements were documented. Where key determinants were not explicitly reported, assumptions were made and retained as sources of uncertainty (and explicitly treated as limitations) in the interpretation of results.

### Modeled exposure

For each company, WCSs were identified according to the CSR. Exposure estimates were generated using the official ART 1.5 web tool (“ART web”) (Ransman et al. 2011; Schinkel et al. 2011; Advanced REACH Tool 2025). While primary results rely on ART web, the study also used the TRanslation of EXposure MOdels (TREXMO 2.0) (Savic et al. 2016, 2020; TREXMO 2025), which serves as a translation and implementation platform for regulatory models, including ART.

In this study, results from ART web were used as the primary outputs for comparison with measured data. The TREXMO 2.0 interfaces were retained only to verify whether the main conclusions depend on the modeling interface. This choice stems from the fact that discrepancies between these platforms may arise from differences in interface implementation, parameter mapping, default settings, versioning, or the translation of inputs. Consequently, we present TREXMO 2.0 exclusively as a secondary sensitivity analysis, avoiding stronger claims on the exact technical origin of the observed differences to ensure the focus remains on the core findings. To minimize coding-related variability, model parameterization was performed using a consistent rule set across all scenarios; nevertheless, assessor judgement remains a recognized source of uncertainty and is acknowledged as a limitation. TREXMO 2.0 was applied in both its “Tier 2” and “TREXMO+” modules. In this context, a “Tier 2” tool refers to a higher-tier exposure model that requires more detailed and specific scenario inputs compared to simpler screening tools. For all tools, the predicted 90th percentile of full-shift exposure concentrations was considered. A specific adjustment was required for the “weight fraction” parameter: while ART web allows the use of predefined categories, TREXMO requires a specific value. Therefore, two runs were performed in TREXMO using the lower and upper bounds of the ART web categories, and their arithmetic mean was used for comparison. Outcomes from TREXMO were systematically compared with those from ART web. The ART parameter inputs are reported in the Supplementary Materials (Table S5a/b/c/d), Section B.

### Measured exposure

Both personal and stationary measurements were included in the study, based on monitoring reports from campaigns conducted in different years by external laboratories using standardized sampling and analytical methods. It was assumed that WCSs had not changed over time. Most samples were below the analytical limit of detection (LOD); results reported as <LOD were retained in the dataset and replaced by the LOD value for descriptive and comparative purposes. The potential impact of this substitution approach on summary statistics and percentile-based comparisons is acknowledged in the interpretation. As the data were generated by third-party laboratories, no additional evaluation or quality assurance/quality control verification could be performed within this study. Given the limited number of measurements available for several scenarios, percentile-based comparisons are interpreted cautiously and are not over-emphasized in the discussion.

### Comparison between outcomes

The comparisons presented here are not intended to establish regulatory compliance decisions; rather, they are used to discuss consistency, conservatism, and uncertainty in model outputs relative to workplace monitoring data. A first comparison was performed between results from TREXMO 2.0 and ART web to evaluate their level of consistency. Subsequently, exposure estimates were compared with measured data for each company to characterize the model's conservatism relative to Cr(VI) summary statistics. Groups of workers—aligned with the concept of a similar exposure group—were defined using information provided in the CSR (ie, CSA and monitoring reports) and assigned to each WCS. Measured exposure data include both personal and stationary samples; these were described separately in the Methods, and comparisons with model outputs were based primarily on personal measurements, with stationary data used only where contextually comparable. A literature review was conducted to identify studies comparing measured and modeled exposure data. The methodological approach followed that of Spinazzè et al. (2017), where predicted values (90th percentile) are considered acceptable if they fall within a factor of five of measured values (0.2 < estimate/measurement < 5). Notably, these acceptability criteria were not validated by subsequent scientific literature; rather, they represented an arbitrary subdivision influenced by a high degree of subjective judgement. In addition, the arithmetic mean and maximum values of the measured data for each group of workers were calculated. To evaluate conservatism, the approach proposed by Lee et al. (2019) was applied, whereby the proportion of estimates lower than measurements define the level of conservatism: low (>25%), medium (11 to 25%), or high (≤10%). Finally, similar WCSs across different companies were compared to analyze how variations in OCs and RMMs influenced the exposure estimates for comparable uses of chromium trioxide.

### Calculation of the risk under REACH and OSH

Because no inhalation DNEL (Derived No Effect Level) or DMEL (Derived Minimal Effect Level) has been established for chromium trioxide, a formal REACH RCR was not calculated. Although occupational exposure limits (OELs) may inform risk assessment in some regulatory contexts, the BOELV for Cr(VI) was not considered equivalent to a REACH DNEL/DMEL in this study. Therefore, ratios based on the BOELV are reported only as OSH-oriented benchmarks for comparative purposes. Risk assessment based on OELs is therefore reported only as OSH-oriented benchmarks for comparison. Instead, the excess risk was derived for each WCS in accordance with RAC/27/2013/06 Rev.1 (ECHA 2013) using exposure estimates obtained from the ART web tool. Calculation assumes a 40-yr working life (8 h/d, 5 d/wk), with total exposure adjusted by modifying factors for activity duration, frequency, and the use of respiratory protective equipment (RPE). It is important to note that such adjustments are not typically practiced within the OSH framework, where exposure concentration is measured out RPE and related to an 8-h shift. This fundamental difference in methodology represents a primary reason why OSH risk assessments and REACH risk assessment are often difficult to compare.

Excess lifetime lung cancer mortality risk was then quantified as defined in Equation 1.


(1) $$\mathrm{Excess}\;\mathrm{lifetime}\;\mathrm{lung}\;\mathrm{cancer}\;\mathrm{mortality}\;\mathrm{risk}=C\_\mathrm{adj}\;(\mu g\;\mathrm{Cr}(\mathrm{VI})/m3)\times 4\times 10^{-3},$$

where C_adj is the concentration adjusted for activity duration, frequency, and, where applicable, RPE. In addition, average exposures and the corresponding excess risk for each group of workers were calculated by considering the worst-case value among the relevant WCSs. Under the OSH framework, risk was expressed as the RCR, defined as the ratio between measured (or estimated) exposure data and the legally established OELs (Equation 2).


(2) $$\begin{array}{rl} \mathrm{RCR}\;= & \mathrm{measured}\;\mathrm{data}\;(\mathrm{or}\;\mathrm{exposure}\;\mathrm{estimate}\;\mathrm{data})/\\ & \mathrm{OEL} \end{array}$$

In detail, the EU Directive 2017/2398 (European Parliament and Council of the European Union 2004), transposed in Italy by Legislative Decree No. 44 of 1 June 2020 (Gazzetta Ufficiale della Repubblica Italiana 2020), sets the limit value at 0.005 mg/m^3^. In this study, the RCR was calculated using personal measurements (RCRp), stationary measurements (RCRs), and exposure estimates from ART (RCRe). Where possible, compliance of measured data with OEL was further assessed according to EN 689:2019, considering the group of workers defined for the combined excess risk. Although excess risk under REACH and RCR under OSH are not directly comparable, both approaches were considered in parallel. Specifically, comparisons were made between RCRp, RCRe, and RCRs, followed by an evaluation of how the different approaches (RCR values, OEL compliance, and excess risk) may lead to diverse conclusions for risk assessment and risk management.

## Results and discussions

### Comparison between different ART tools

As shown in Table 2, a generally good consistency was observed between ART web and the alternative ART interfaces. Across the four companies, 3 out of 35 WCSs using TREXMO produced results that differed from ART web. In these cases, the ratios between the TREXMO tool and ART web indicated discrepancies of up to two orders of magnitude. Overall, TREXMO exhibited lower exposure estimates when compared with ART web. TREXMO+, by contrast, showed a high level of consistency with ART web across all cases. In the scientific literature, only a few studies have compared TREXMO with ART (Savic et al. 2018, 2019, 2020). A relevant example is Savic et al. (2020), which directly evaluated TREXMO+ against conventional models (ART, Stoffenmanager, ECETOC TRAv3). The study suggested that TREXMO+ was less biased and more accurate, with predictions typically within a factor of 2 to 3 of measurements, compared to factors of 2 to 14 for the conventional models. However, these findings were based on a limited dataset, particularly for powders and solids, which constrains broader validation. In the present study, TREXMO+ produced outcomes largely comparable with ART web, and most TREXMO results also showed close alignment. For this reason, comparisons of TREXMO+, TREXMO, and measured data were considered consistent with those obtained from ART web. A first comparison was carried out between the ART web outcomes, considered as the reference for this study, and those obtained with TREXMO 2.0, analyzing each company and WCS separately. More detailed results and comprehensive comparisons for each company are provided in the Supplementary Materials, Section B (Table S6a/b/c/d, Section C).

**Table 2:** Comparison between results of ART tools (TREXMO and TREXMO+) and ART web for each WCS. The ratio between the estimate of ART tools and ART web defines the level of consistency of the ART tools compared to ART web. (N WCSs: number of Worker Contributing Scenarios).

Company	N WCSs	TREXMO +	TREXMO	AnRT tools/ART web	Comment
A	8	8/8	7/8 WCS2	1.00 × 10^−1^ TREXMO	Low consistency
B	8	8/8	8/8	−	High consistency
C	8	8/8	7/8 WCS4	1.00 × 10^−2^ TREXMO	Low consistency
D	11	11/11	10/11 WCS11	2.10 × 10^−1^ TREXMO	Low consistency

### Comparison of ART Web exposure estimates and measured data: level of consistency and conservatism

ART web exposure estimates (90th percentile, 95% confidence interval) were compared with measured 8-h time-weighted average (TWA) exposures for each WCS across the four companies (Table 3). Results from TREXMO were not included here, as their outcomes were generally consistent with ART web. Personal and stationary data were analyzed separately. For each WCS, the 90th percentile of the measured series was calculated, together with the predicted-on-measured ratio, following (Spinazzè et al. 2017). Average mean and maximum measured values are reported in the Supplementary Materials (Table S7a/b/c/d, Section C). In line with Spinazzè et al. (2017), predictions were considered acceptable if within a factor of 5 of the measurements (0.2 < estimate/measurement < 5). Overall, ART tended to get a low level of consistency—or lower exposure estimates—in at least half of the WCSs, both for personal and stationary data, except for personal measurements in Company A. Specifically, a low level of consistency occurred in 22 of 34 WCSs (65%) with personal data and in 25 of 35 WCSs (71%) with stationary data. Company C showed systematic low consistency across all WCSs, whereas Company B displayed a mixed outcome, with about half of the WCSs got a low level of consistency and half within the acceptable range. In most cases, the levels of consistency were up to two orders of magnitude compared with measured values; only in Company A (stationary data) were discrepancies limited to about one order of magnitude. Attempts to compare “similar” WCSs across different companies (eg A versus D, B versus C) were generally inconclusive. Although the uses of chromium trioxide appeared comparable, differences in input parameters such as RMMs, activity duration, containment level, weight fraction, and exposure field led to diverging results. Only a few cases (eg WCS 3 to 5 and 8 to 6 for A to D, WCS 4 to 8 for B to C) showed comparable outcomes despite parameter differences. Although in this study many WCSs were defined in a comparable way, it should be emphasized that four different companies and two distinct uses of chromium trioxide were analyzed. The exposure context in each company may vary substantially depending on several factors, such as the size and layout of the production line(s), the physical state of chromium trioxide in refilling and rinsing WCSs (solid or liquid), the implemented RMMs, and the OCs. Furthermore, the selection of ART input parameters may be influenced by the operator's judgement and experience with the model, as well as by the inherent subjectivity of some parameters. Another limitation lies in the comparability between measured and estimated data. Under the REACH tiered approach, exposure assessment is primarily model-based, and the definition of exposure scenarios may remain too generic compared to actual workplace conditions. In contrast, measurements reflect daily worker activities, which in small- and medium-sized companies often cover multiple WCSs, some of which are characterized by low frequency or short duration. In this study, it was assumed that measurements correspond to a workday during which all tasks defined in the WCSs were performed. However, this assumption may have led to a forced alignment between measured and modeled data. This consideration highlights the crucial role of the assessor's expertise, both in selecting and applying exposure model parameters and in interpreting the resulting estimates against real workplace conditions, particularly within the REACH framework (Schinkel et al. 2014; Landberg et al. 2015; Lamb et al. 2017; Savic et al. 2019).

**Table 3:** Level (N/total) of consistency between ART exposure estimates and measured data.

Company	Type of measured data	Good	Lower	Higher
A	Personal	4/7	2/7	1/7
	Stationary	2/8	5/8	1/8
B	Personal	4/8	4/8	0/8
	Stationary	4/8	4/8	0/8
C	Personal	0/8	8/8	0/8
	Stationary	0/8	8/8	0/8
D	Personal	3/11	8/11	0/11
	Stationary	3/11	8/11	0/11

Concerning the level of conservatism of ART, the percentage of exposure estimates lower than measured data was used as the evaluation criterion defined above (low conservatism: >25%; medium: 11 to 25%; high: ≤10%) (Lee et al. 2019). As summarized in Table 4, when all data (personal and stationary) were aggregated at the company level, ART generally showed a low level of conservatism. However, when results were examined at the level of individual WCSs, all categories of conservatism were observed (Tables S8a/b/c/d, Supplementary Materials, Section C), highlighting substantial variability across tasks and contexts. It should also be noted that the number of measurements available per WCS was often limited, which reduces the strength of the conclusions. For example, in Companies A and C, personal datasets included only 3 to 9 and 2 to 5 measurements, respectively. A larger dataset was available for Company D (18 personal and 20 stationary measurements), but these were associated with 12 WCSs belonging to a single group of workers. Consequently, in some cases, the measured data are representative of the overall daily exposure of one worker group rather than of individual tasks, and the conservatism analysis should be interpreted with caution. To date, no evidence is available in the scientific literature regarding the reliability of ART for plating activities or for assessing exposures to Cr(VI). However, several studies have investigated its performance and reliability in other industrial contexts. Overall, ART is described here as a widely used and extensively evaluated higher-tier exposure model; however, its performance is context-dependent and may vary across tasks, substances, and the quality of input information (Spinazzè et al. 2017, 2019; Lee 2023; Chiurtu et al. 2024). Nevertheless, several studies have highlighted that ART may either underestimate or overestimate exposures depending on the exposure scenario.

**Table 4:** Level of conservatism of ART. E: estimated exposure data; M: measured data; n: number.

Company	E>M (n)	%	E<M (n)	%	Level of conservatism
Personal					
A	9/27	33	18/27	67	Low
B	17/72	23	55/72	77	Low
C	0/33	0	33/33	100	Low
D	52/198	26	146/198	74	Low
Stationary					
A	12/30	40	18/30	60	Low
B	20/72	28	52/72	72	Low
C	0/60	0	60/60	100	Low
D	46/220	21	174/220	79	Low

According to Riedmann et al. (2015), these discrepancies are largely driven by the model's high sensitivity to the source compartment, which accounts for approximately 75% of the total exposure range; consequently, even minor inaccuracies in defining this critical parameter can result in an exposure estimate variance of up to 22 orders of magnitude. Underestimations were observed in cases involving pesticide use, handling of liquids with vapor pressure > 10 Pa (except for activities on relatively undistributed surfaces), and tasks in the wood industry (Spinazzè et al. 2019). Additional examples include solvent cleaning operations (Lee et al. 2019), dumping and mixing of nanopowders (eg, TiO_2_, Al_2_O_3_, SiO_2_) (Bekker et al. 2016), as well as activities with powders, vapors, solids (Savic et al. 2017), and volatile liquids with vapor pressures > 10 Pa (Lee et al. 2019). Conversely, overestimations have been reported in ceramic material packing, in the same activities mentioned above but at lower exposure levels, and in degreasing, varnishing, and painting tasks with organic solvents. Furthermore, the study by Landberg et al. (2017) confirmed a generally good consistency between ART predictions and measured data across a range of sectors, including wood, printing, foundry, spray painting, flour milling, chemical, and plastic molding industries. In that study, measured exposures exceeded ART estimates in only one case, while spray painting with toluene showed particularly high consistency.

### RCR under OSH and under REACH

REACH and OSH offer complementary approaches to occupational exposure: the former focuses on risk characterization through exposure scenarios, while the latter emphasizes measurement-based control. However, under both regimes, the actual control of risks must be validated via workplace measurements, with national authorities ensuring REACH compliance through field monitoring. As mentioned before, since chromium trioxide is identified by ECHA as a nonthreshold carcinogen, no DNEL or DMEL has been defined for inhalation exposure. Consequently, an RCR under REACH could not be calculated. Instead, following RAC/27/2013/06 Rev.1, the excess lifetime lung cancer mortality was calculated assuming 40 yr of exposure (8 h/d, 5 d/wk), with adjustments for frequency, duration, and RPE. Table S9 in Supplementary Materials reports the excess risk under REACH and the RCR under OSH, while combined exposures for all companies and WCSs are shown in Table 5. The exposure modifying factors are presented in Table S10 in Supplementary Materials, Section D. For these calculations, only ART web exposure estimates were considered. For comparative purposes, the exposure estimates from ART were also used to derive an RCR as the ratio between exposure estimate and the Binding Occupational Exposure Level Value (BOELV) of 0.005 mg/m^3^ defined by EU Directive 2022/431. Two additional RCR were calculated using personal and stationary measured data. Table 6 shows the mean values for each company. In all cases, RCRp, RCRs, and RCRe were <1, suggesting that risks could be considered acceptable under a standard OSH approach. Specifically, the mean RCRp values were <0.1 in Company A, 0.17 in Company B, 0.06 in Company D, and ranged between 0.35 and 0.50 in Company C. In most WCSs, RCRe values were lower than RCRp, but some exceptions emerged: one WCS in Company B and six in Company C showed RCRe values of 0.72 to 0.76, while corresponding RCRp values were 0.17 and 0.35 to 0.45, respectively. These cases, although still <1, indicate WCSs that would benefit from closer surveillance. The RCRs were generally comparable with RCRp, except in Company D (RCRs three times higher but still <0.2) and in Company C (RCRs in WCS7-8 about half the RCRp).

**Table 5:** Combined WCSs, excess risk, and RCR.

Company	Type of workers	Combined WCSs	Excess risk (exposure estimates)	Preliminary test	Statistical test
A	Conductor of the plating line	1 + 2 + 3 + 4 + 5 + 8	1.28 × 10^−04^	In compliance	…
A	Load/unload operators	7	9.20 × 10^−05^	…	Not in compliance
A	Conductor of the treatment plant	1 + 2 + 3 + 4 + 5 + 6 + 8	1.28 × 10^−04^	No personal measured data available. Stationary data insufficient for the preliminary test	
B	Functional chrome plating	1 + 2 + 3 + 4 + 5 + 6 + 7 + 8	2.80 × 10^−03^	…	Not in compliance
C	Conductor of the process	1 + 2 + 3 + 4 + 5 + 6	4.80 × 10^−05^	In compliance	…
C	Supervisor of the plant	6	1.04 × 10^−05^	…	In compliance (stationary data)^a^
C	Laboratory operator	7 + 8	3.55 × 10^−06^	…	In compliance (stationary data)^a^
D	Plating line technician	1 + 2 + 3 + 4 + 5 + 6 + 7 + 8 + 9 + 10 + 11 + 12	1.76 × 10^−03^	…	In compliance

^a^Personal data insufficient for the preliminary test

**Table 6:** Mean of RCR divided in personal measured data, stationary measured data, and estimated exposure and the excess risk.

Company	RCRp	RCRe	RCRs	Excess risk
A	0.01	0.047 (0.00064 to 0.32)	0.016 (0.01 to 0.022)	2.96 × 10^−05^ (1.60 × 10^−10^ to 1.28 × 10^−04^)
B	0.17	0.13 (3.6 × 10^−4^ to 0.72)	0.17	3.86 × 10^−04^ (5.28 × 10^−09^ to 2.80 × 10^−03^)
C	0.43 (0.35 to 0.50)	0.67 (0.40 to 0.76)	0.30 (0.25 to 0.31)	1.33 × 10^−05^ (9.57 × 10^−07^ to 1.04 × 10^−05^)
D	0.06	0.034 (2 × 10^−6^ to 0.19)	0.18	3.26 × 10^−04^ (2.56 × 10^−09^ to 1.76 × 10^−03^)

### Implications for risk assessment and management

Although RCR and excess risk cannot be directly compared, insights can be gained by considering both. The excess risk, as defined in RAC/27/2013/06 Rev.1, is positioned within the “traffic-light” framework: the boundary for tolerable risk corresponds to an additional cancer risk of 4 × 10^−3^ while the boundary for acceptable risk is 4 × 10^−5^ (BAuA, 2013). The acceptable and tolerable risk values are reported as policy benchmarks used in specific regulatory contexts and are included here for comparative interpretation, not as universally applicable EU-wide standards. In this study, excess risks were consistently below the tolerable threshold but generally closer to it than to the acceptable benchmark. Notably, WCS5 in Company B and WCS8 and WCS9 in Company D were of the same order of magnitude as the tolerable threshold even though their RCR remained well below 1 (≤0.17 to 0.18). Conversely, some WCSs with RCRe values close to 1 (eg, 0.76) were associated with excess risks 2 to 4 orders of magnitude lower than the tolerable risk, underlining the different perspectives provided by the two approaches. Application of EN 689:2019 allowed verification of compliance with the OEL through preliminary and statistical tests. Results (Table 4) showed cases of noncompliance despite RCR <1. For instance, in Company A “Load and unload operators” did not comply with the OEL, while for “Conductor of the treatment plant” no valid test could be performed due to the lack of sufficient personal data. In Company B, compliance was also not achieved for the single group of workers considered (functional chrome plating). Company C showed insufficient personal data for some groups, requiring the use of stationary measurements instead. In these cases, further assessment and risk management measures are needed to ensure adequate protection.

### REACH–OSH interactions

Overall, these findings highlight how REACH and OSH provide complementary perspectives on risk for workers. While REACH focuses on substance—and use—related risks along the supply chain, OSH emphasizes workplace-specific compliance with OELs and good practice. Evidence from the AfA process indicates that REACH can significantly strengthen OSH by imposing monitoring and other conditions as part of authorization and registration. Monitoring is the most frequent requirement, ensuring annual verification of the effectiveness of OCs and risk management measures. This complementarity was also emphasized at the 5th German REACH Congress, where REACH was recognized as a driver for substitution and stricter risk management, aligning closely with the OSH “STOP” hierarchy of measures (Substitution, Technical, Organizational, and Personal). Coordinated implementation of REACH and OSH instruments therefore supports a more effective and comprehensive strategy for chemical risk management in occupational environments. Thus, under OSH, more efforts are needed to better manage risks and to properly evaluate exposure. Since worker health protection is a common goal of both REACH and OSH, synergies occur in pursuing it and can be used to improve worker protection.

### Additional considerations on model-related limitations and interpretation

From a study-design perspective, this work was not intended to provide a general validation of ART (or of occupational exposure models as a class), nor to resolve the broader theoretical debate on model architectures. It was designed as a case study to compare model-based and measurement-based exposure assessment outcomes for chromium trioxide under real REACH authorization and OSH conditions.

A comparison with Tier 1 screening tools was outside the scope of the present case study. In the context of carcinogenic substances such as chromium trioxide, such tools should not be regarded as a substitute for workplace measurements and context-specific assessment. Therefore, the observed discrepancies between modeled estimates and measured values should be interpreted primarily as evidence of the importance of scenario definition, input quality, assessor competence, and contextual interpretation, rather than as proof of universal model inadequacy (Fransman 2017; Spinazzè et al. 2019; Schlüter and Spinazzè 2023). More broadly, occupational exposure models used in regulatory contexts remain valuable but imperfect tools, with recognized limitations related to transparency of assumptions, parameter interpretation, interassessor variability, and uneven performance across scenarios (Savic et al. 2018, 2019; Jones 2022; Schlüter et al. 2022). Recent contributions have also highlighted the need to strengthen transparency, quality criteria, and uncertainty communication in REACH-oriented modeling practice (Koivisto and Jayjock 2025). In our view, these issues support a fit-for-purpose approach rather than a “models versus measurements” opposition: models are useful to structure scenarios and prioritize action, whereas workplace measurements remain essential for verification, compliance demonstration, and refinement of risk management decisions in both REACH and OSH contexts.

## Conclusions

This study compared modeled exposure estimates and measured data for Cr(VI) in four Italian plating companies, with the aim of analyzing how risk is characterized and managed under the REACH and OSH frameworks. The study revealed important differences between the two approaches, especially regarding the role of exposure models and workplace monitoring. Under REACH, exposure assessment largely relies on models following the tiered approach as reported in the ECHA's R14 guidance, while monitoring often is introduced as a condition of authorization. Conversely, OSH legislation is firmly rooted in workplace monitoring, with models having a supporting role, for example in basic exposure characterization under EN 689:2019. In this study, the comparison between exposure estimates and measured data revealed how a low level of consistency might occur in some cases by up to two orders of magnitude. In this perspective, these discrepancies should be interpreted primarily as evidence of the critical importance of scenario definition, input quality, and contextual interpretation, rather than as proof of universal model inadequacy. This highlights the limitations of relying solely on models when assessing “safe use” conditions, and the importance of assessor expertise in selecting input parameters and interpreting outcomes, addressing known limitations such as interassessor variability and the lack of transparency in model assumptions. In this study, measured data allowed the calculation of RCR, which was always below 1, suggesting compliance with OEL, although EN 689:2019 compliance testing identified some cases of nonconformity, putting attention on them. This underlines how the OSH framework can provide a more context-specific picture of daily worker exposure. The comparison also revealed potential complementarities between the REACH and OSH frameworks. While OSH focuses on ensuring continuous compliance and safe working conditions at the workplace level, REACH authorizations often impose stricter requirements—such as mandatory exposure monitoring and enhanced RMMs—that reinforce OSH provisions and promote the “STOP” hierarchy of controls, particularly through substitution and technical measures. Therefore, a coordinated and complementary application of REACH and OSH instruments can lead to more consistent and effective chemical risk management in occupational settings. This complementarity has also been highlighted in recent analyses showing that the AfA process under REACH frequently results in substantial improvements in OSH. These improvements mainly derive from the implementation of monitoring obligations recommended by RAC and SEAC or mandated by the European Commission. Monitoring has emerged as the most common protective measure, serving to verify the effectiveness of OCs and RMMs and typically requiring annual review throughout the authorization period. Moreover, discussions at the 5th German REACH Congress emphasized REACH's dual role as both a driver of substitution of hazardous substances and an enabler of adequate control of risks associated with SVHCs. In conclusion, while this work does not aim to provide a general validation of exposure models, it supports a “fit-for-purpose” approach rather than a “models versus measurements” opposition. Exposure models are useful tools under both frameworks to structure scenarios and prioritize actions, but their reliability depends heavily on contextualization with workplace data and on the competent handling by users of the models (however also workplace monitoring needs to be performed competently). Integrating modeled exposure estimates with systematic monitoring, and aligning REACH and OSH requirements, represents a more robust and consistent strategy to protect worker health from exposures to carcinogens.

## Supplementary Material

wxag046_Supplementary_Data

## Data Availability

The data underlying this article are available in the article and in its online supplementary material.
