# Supplementary material for: Comparison of measured and modeled data for carcinogen workplace exposure assessment: a case study on chromium trioxide under REACH and OSH frameworks
Source: Ann Work Expo Health. 2026 Jun 22;70(5):wxag046. doi: 10.1093/annweh/wxag046 (PMC13285727; doi:10.1093/annweh/wxag046)
Supplement: wxag046_Supplementary_Data [file wxag046_supplementary_data.pdf]

Comparison of measured and modelled data for carcinogen workplace exposure assessment: a case study on Chromium Trioxide under REACH and OSH Frameworks.

Carolina Zellino[1], Urs Schlüter[2], Raffaella Wree[2], Ann Carolin Dumke[2], Gudrun Walendzik[2], Chiara Marelli[3], Eleonora Pagani[1], Rocco Loris Del Vecchio1, Alessio Carminati[1], Sabrina Rovelli[1], Giacomo Fanti[4], Francesca Borghi[5], Andrea Cattaneo[1], Andrea Spinazzè[1], Domenico Maria Cavallo[1].

[1] Department of Science and High Technology, University of Insubria, Como, Italy.

[2] Federal Institute for Occupational Safety and Health, Dortmund, Germany.

[3] Team Mastery, Como, Italy.

[4] Department of Clinical and Community Sciences – DISCCO - "Clinica Del Lavoro Luigi Devoto", University of Milan, Milano, Italy.

[5] Department of Medical and Surgical Sciences, University of Bologna, Bologna, Italy.

SUPPLEMENTARY MATERIAL

SECTION "A" - WCSs

Table S1a - Company A - Contributing Scenarios presented in the Use.

Contributing scenario	PROC	Name of the scenario	Description
WCS 1	26	Refilling of the plating bath with Chromium Trioxide	The conductor of the process manually lifts the drum of CrO_3 (25 Kg) at the level of the tank surface (about 1 m above the floor in the corridor located near the plating line) and totally emptied
WCS 2	8b	Rinsing of Chromium Trioxide drum with water	After the decanting step, the conductor of the process rinses the drum with water, using a hose, and pours the solution in the Chromium containing tank
WCS 3	8b	Refilling of the Chromium trioxide tank with reactants	The conductor of the process adds several other reactants (i.e., $H2SO_4$, catalyst, mist suppressant) in the tank containing CrO_3 to create the right environment for Chromium deposition
WCS 4	13	Supervision/Conduction of the plating line	The conductor of the process adds reactants to the tanks, performing general maintenance and doing density measurements. Workers spend most of the time controlling the process from a remote position and only, when necessary, reach the plating line for doing routine controls on the line
WCS 5	28	General maintenance	The conductor of the process cleans the tanks containing CrO_3, maintains the lip extraction system and does minor activities related to the good working of the process
WCS 6	3	Supervision/Conduction of the effluent treatment plant	The Conductor of the wastewater plant controls the liquid flows, the addition of reducing, checks the redox and pH probes by cleaning and calibrating them
WCS 7	21	Load and unload of the components	Line operator loads the components to be electroplated on the jigs and unloads them afterwards. A visual control of finished parts is done. The nearest workplace is about 5 meters away from the plating line
WCS 8	15	Density measurement	The conductor of the process measures the density of the bath by immersing the densimeter in the plating solution

Table S1b - Company A - Operational Conditions and Risk Management Measures.

Contributing scenario	Duration (mins)	Frequency* (day/year)	LEV used	RPE used	Other RMMs	Ventilation
WCS 1	5	88	On-tool extraction (90% reduction)	Respirator APF20, efficiency = 95 %	Gloves resistant to acid + goggles + working suit	3 ACH
WCS 2	10	88	On-tool extraction (90% reduction)	Respirator APF20, efficiency = 95 %	Gloves resistant to acid + goggles + working suit	3 ACH
WCS 3	2	220	On-tool extraction (90% reduction)	FFP1 Mask APF10 efficiency = 90 %	Gloves + goggles, working suit	3 ACH
WCS 4	10	220	On-tool extraction (90% reduction)	FFP1 mask APF10 efficiency = 90 %	Gloves + goggles, working suit	3 ACH
WCS 5	120	1	On-tool extraction (90% reduction)	Respirator APF20, efficiency = 95 %	Gloves resistant to acid + goggles + working suit, anti-acid boots	3 ACH
WCS 6	5	44	High level containment (99.90% reduction)	FFP1 mask APF10 efficiency = 90 %	Gloves + goggles, working suit	3 ACH
WCS 7	480	220	On-tool extraction (90% reduction)	-	Gloves, working suit	3 ACH
WCS 8	2	220	On-tool extraction (90% reduction)	FFP3 mask APF10 efficiency = 90 %	Gloves + goggles	3 ACH

* 250 workdays/year, APF: Assigned Protection Factor

Table S2a - Company B - Contributing Scenarios presented in the Use.

Contributing scenario	PROC	Name of the scenario	Description
WCS 1	26	Refilling of the chrome baths with Chromium Trioxide	The worker manually carries out the drum of CrO_3 in front of the bath to be filled, lifts at the level of the tank surface and empties it. The current of the baths is off during refilling of the baths.
WCS 2	8b	Rinsing of Chromium Trioxide drums with water	The worker rinses the drum with water using a hose. The rinsing water is subsequently poured into the bath.
WCS 3	5	Refilling of the chrome baths with the catalyst	The worker adds additives in the Cr baths<br>Sulphuric acid is added to the Cr baths only when the baths are renovated.
WCS 4	13	Sampling of the chrome baths for subsequent laboratory analysis and in situ measurement of the chrome bath density	The worker measures the density of Cr baths in situ using a densimeter. The measurement of the density is performed when the current is off.
WCS 5	21	Loading and unloading of the articles on/from the jigs and quality check	The worker loads the articles in the Cr baths when the current is turned off. Articles heavier than 8 Kg are loaded in the "Flag" crane system, approximately 4 meters from the chrome baths. The articles are then immersed in the bath. Smaller articles (lighter than 8 Kg) are placed directly in the chrome baths. After plating and washing, the treated articles must be unloaded from the jigs and quality check is performed.
WCS 6	28	Supervision/conduction of the plating line and daily maintenance of the plating line	The worker approaches the chrome baths for control and maintenance of the line.
WCS 7	13	Washing of the treated articles	At the end of the chrome plating process, the worker pre-washes the article with gentle hose in the Cr baths and then moves it to the washing baths.
WCS 8	28	Low frequency maintenance of the plating line	Besides the daily maintenance of the functional chrome plating line, the operator keeps the plating line, and the lip extraction system cleaned. This scenario does not consider the emptying of the chrome baths, which is performed only once every five years by a specialized and accredited company

Table S2b - Company B - Operational Conditions and Risk Management Measures.

Contributing scenario	Duration (mins)	Frequency* (day/year)	LEV used	RPE used	Other RMMs	Ventilation
WCS 1	10	48	Fixed capturing hoods	respirator APF20 (efficiency = 95 %)	Gloves resistant to acid + goggles + working suit + working shoes	3 ACH
WCS 2	10	48	Fixed capturing hoods	respirator APF20 (efficiency = 95 %)	Gloves resistant to acid + goggles + working suit + working shoes	3 ACH
WCS 3	10	6	On-tool extraction	respirator APF20 (efficiency = 95 %)	Gloves resistant to acid + goggles + working suit + working shoes	3 ACH
WCS 4	10	48	On-tool extraction	respirator APF20 (efficiency = 95 %)	Gloves resistant to acid + goggles + working suit + working shoes	3 ACH
WCS 5	300	250	On-tool extraction		Working suit, working shoes	3 ACH
WCS 6	20	250	On-tool extraction	respirator APF20 (efficiency = 95 %)	Gloves resistant to acid +goggles + working suit + working shoes	3 ACH
WCS 7	20	250	On-tool extraction	respirator APF20 (efficiency = 95 %)	Gloves resistant to acid +goggles + working suit + working shoes	3 ACH
WCS 8	60	2	On-tool extraction	respirator APF20 (efficiency = 95 %)	Gloves resistant to acid + goggles + working suit + working shoes	3 ACH

* 250 workdays/year, APF: Assigned Protection Factor

Table S3a - Company C - Contributing Scenarios presented in the Use.

Contributing scenario	PROC	Name of the scenario	Description
WCS 1	8b	Refilling of the electroplating line with Chromium Trioxide solution	The conductor of the process adds the solution of Chromium Trioxide in the machine through a closed system of tubes that are directly connected to the electroplating line. The addition is managed by a dosing pump connected to a remote control. The LEV system is maintained active for 24h/day. During this task, the conductor of the process is exposed to the solution of Cr(VI) only during the opening of the tank and connection of the tubes to the machine.
WCS 2	13	Rising of the treated pieces after the electroplating process	Before being removed from the electroplating line, the conductor of the process rinses the treated piece with water using a pipe.
WCS 3	13	Load/unload of the cylinders	The conductor of the process manually loads the piece to be treated inside the machines onto appropriate supports. The current is not applied. The plating process is started with a remote control when the machine is totally closed. The machines in fact are provided with doors that can be opened to load or to unload the components. CrO_3 solution is pumped through pipes inside the cylinder bore when the process starts and the current is applied.
WCS 4	13	Rinsing of the treated cylinders in rising tanks	The conductor of the process rinses the treated article with water using a pipe inside the plating line.<br>The rinsing phase is also helped using brushes.
WCS 5	28	General maintenance	The conductor of the process performs the general maintenance i.e., cleaning of the electroplating machines, maintenance of the aspiration system and minor activities related to the good working of the process.
WCS 6	13	Supervision of the electroplating plant	The supervision of the electroplating plant is performed by the conductor of the line and by the supervisor of the plant.
WCS 7	8b	Supervision/Conduction of the effluent treatment plant	The laboratory operator keeps the effluent treatment plant running without directly handling Cr(VI) containing liquids neither approaching the tanks where the wastewater with Cr(VI) is contained. All the wastewater is collected and transferred to the effluent treatment plant by means of a closed system.
WCS 8	15	Sampling and density measurement	The laboratory operator regularly performs density and ionic HPLC measurements. The current is not applied.

Table S3b - Company C - Operational Conditions and Risk Management Measures.

Contributing scenario	Duration (mins)	Frequency* (day/year)	LEV used	RPE used	Other RMMs	Ventilation
WCS 1	15	94	On-tool extraction (90.00 % reduction)	Respirator APF10, efficiency = 90 %	Gloves, boots and working suit resistant to acid resistant to acid + goggles	10 ACH
WCS 2	20	220	On-tool extraction (90.00 % reduction)	Respirator APF10, efficiency = 90 %	Gloves, boots and working suit resistant to acid resistant to acid + goggles	10 ACH
WCS 3	30	220	On-tool extraction (90.00 % reduction)	no	gloves, boots and working suit resistant to acid resistant to acid + goggles	10 ACH
WCS 4	30	220	No localized controls (0.00 % reduction)	no	Gloves, boots and working suit resistant to acid resistant to acid + goggles	10 ACH
WCS 5	75	220	On-tool extraction (90.00 % reduction)	Respirator APF10, efficiency = 90 %	Gloves, boots and working suit resistant to acid resistant to acid + goggles	10 ACH
WCS 6	70	220	On-tool extraction (90.00 % reduction)	FFP3 mask APF10 efficiency = 90 %	Gloves + goggles + working suit	10 ACH
WCS 7	50	220	No localized controls (0.00 % reduction)	FFP3 mask APF10 efficiency = 90 %	Gloves + goggles + working suit	Only good natural ventilation
WCS 8	15	30	On-tool extraction (90.00 % reduction)	no	Gloves + goggles + working suite	1 ACH

* 220 workdays/year, APF: Assigned Protection Factor

Table S4a - Company D - Contributing Scenarios presented in the Use.

Contributing scenario	PROC	Name of the scenario	Description
WCS 2	26	Refilling of the line tanks with Chromium Trioxide liquid form	The plating line technician adds the solution into the plating bath with an automatic system with the use of a pump that transfers directly the solution from IBC container to the plating tank. The plating line is switched off, but the LEV is kept operating.
WCS 3	26	Refilling of the line tanks with Chromium Trioxide solid form	If necessary, a manual drum clamp is attached to the drum that will be attached to the chain of a hoist. The drum is lifted up near the plating tank and the substance is then added to the bath. The plating line is switched off, but the local exhaust ventilation is still operating.
WCS 4	8b	Rinsing of Chromium Trioxide drum with water	After decanting Chromium Trioxide into the baths, the plating line technician washes the drum with water using a hose. This activity is repeated three times and the water containing Cr(VI) is directly spilled into the containment basin.
WCS 5	8b	Refilling of the line tanks with other components (sulphuric acid and mist suppressant)	The plating technician manually adds concentrated sulphuric acid (98%) in plastic drums (25 L) into the bath. The plating line is switched off, but the LEV is still operating.
WCS 6	15	Sampling and density measure of the plating bath	The plating technician performs density and measure samples. The plating line is switched off, but the LEV is still operating.
WCS 7	21	Load and unload of the plates	The plating technician manually fixes the stainless-steel plates with jigs onto a carrier at the preparation area located at about 10 m from the plating line. The process consists of moving stainless steel plates fixed on a bridged crane and dipping them in the various tanks in a semi-automatic way using a remote control.
WCS 8	13	Electroplating of press plates	After several pre-treatment steps, where the plate is lifted in different baths (degreasing bath, rinsing bath), it is lowered into the chrome bath and the crane hook is detached from the carrier.
WCS 9	13	Dechroming of plated stainless-steel press plates	The plating technician navigates the plates to and from the baths via a remote control.
WCS 10	13	Rinsing of the plated plates with watering pipe	The plating technician rinses the treated stainless-steel plates with water when they are still hanging on the bridged crane using a watering pipe.
WCS 11	13	Waste water management	The plating technician is responsible for keeping the effluent treatment plant running. NO directly handling Cr(VI) containing liquids neither approaching the tanks (fully automatic and enclosed process).
WCS 12	3	General maintenance including cleaning of the plating tanks	The plating technician cleans the plating area and tanks, maintains the lip extraction system and keeps minor activities related to the good working of the process.

Table S4b - Company D - Operational Conditions and Risk Management Measures.

Contributing scenario	Duration (mins)	Frequency* (day/year)	LEV used	RPE used	Other RMMs	Ventilation
WCS 2	10	20	On-tool extraction (90.00 % reduction)	FFP3 mask APF10 efficiency = 90 %	Gloves, boots and working suit resistant to acid resistant to acid + goggles	10 ACH
WCS 3	15	40	On-tool extraction (90.00 % reduction)	FFP3 mask APF10 efficiency = 90 %	Gloves, boots and working suit resistant to acid resistant to acid + goggles	10 ACH
WCS 4	5	40	On-tool extraction (90.00 % reduction)	FFP3 mask APF10 efficiency = 90 %	Gloves, boots and working suit resistant to acid resistant to acid + goggles	10 ACH
WCS 5	5	1	On-tool extraction (90.00 % reduction)	FFP3 mask APF10 efficiency = 90 %	Gloves, boots and working suit resistant to acid resistant to acid + goggles	10 ACH
WCS 6	5	250	On-tool extraction (90.00 % reduction)	FFP3 mask APF10 efficiency = 90 %	Gloves, boots and working suit resistant to acid resistant to acid + goggles	10 ACH
WCS 7	30	250	Fixed capturing hood (90.00 % reduction)	no	Gloves + working suit	10 ACH
WCS 8	300	250	On-tool extraction (90.00 % reduction)	no	Gloves + goggles + working suit	10 ACH
WCS 9	300	250	On-tool extraction (90.00 % reduction)	no	Gloves + goggles + working suit	10 ACH
WCS 10	60	250	Fixed capturing hood (90.00 % reduction)	no	Gloves + working suit	10 ACH
WCS 11	10	250	High level containment (99.90 % reduction)	FFP3 mask APF10 efficiency = 90 %	Gloves + goggles	1 ACH
WCS 12	30	2	On-tool extraction (90.00 % reduction)	FFP3 mask APF10 efficiency = 90 %	Gloves, boots and working suit resistant to acid resistant to acid + goggles	10 ACH

* 250 workdays/year, APF: Assigned Protection Factor

SECTION B - Input of ART parameters

Table S5a - Company A - ART parameters. NF: Near-field exposure; FF: Far-field exposure; na: not available.

	WCS1	WCS2	WCS3	WCS4	WCS5	WCS6	WCS7	WCS8
**Total duration (mins)**	5	10	2	10	120	5	480	2
**Non exposure period (mins)**	475	470	478	470	360	475	0	478
**Field exposure**	NF	NF	NF	NF	NF	NF	FF	NF
**OC: substance emission potential**								
	**WCS1**	**WCS2**	**WCS3**	**WCS4**	**WCS5**	**WCS6**	**WCS7**	**WCS8**
**Substance product type**	Powders, granules or pelletised material	Powders, granules or pelletised material	Powders dissolved in a liquid or incorporated in a liquid matrix	Powders dissolved in a liquid or incorporated in a liquid matrix	Powders dissolved in a liquid or incorporated in a liquid matrix	Powders dissolved in a liquid or incorporated in a liquid matrix	Powders dissolved in a liquid or incorporated in a liquid matrix	Powders dissolved in a liquid or incorporated in a liquid matrix
**Liquid matrix weight fraction**	na	na	Substantial	Substantial	Substantial	Very small	Substantial	Substantial
**Viscosity**	na	na	Low	Low	Medium	Low	Low	Low
**Dustiness**	Granules, flakes or pellets	Granules, flakes or pellets	na	na	na	na	na	na
**Moisture content**	Dry product (< 5 % moisture content)	Dry product (< 5 % moisture content)	na	na	na	na	na	na
**Powder weight fraction**	Pure material	Pure material	na	na	na	na	na	na
**OC: Activity emission potential**								
	**WCS1**	**WCS2**	**WCS3**	**WCS4**	**WCS5**	**WCS6**	**WCS7**	**WCS8**
**Activity class**	Falling powders	Handling of contaminated solid objects or paste	Activities with relatively undisturbed surfaces (no aerosol formation)	Activities with agitated surfaces	Activities with relatively undisturbed surfaces (no aerosol formation)	Activities with relatively undisturbed surfaces (no aerosol formation)	Activities with agitated surfaces	Activities with relatively undisturbed surfaces (no aerosol formation)
**Situation**	Transferring 10 – 100 kg/minute	Handling of objects with limited residual	Open surface 1 - 3 m²	Open surface 1 - 3 m²	Open surface 1 - 3 m²	Open surface 1 - 3 m²	Open surface 1 - 3 m²	Open surface 1 - 3 m²

		dust (thin layer visible)						
**Hendling type**	Careful handling involves workers showing attention to potential danger, error or harm and carrying out the activity in a very exact and thorough (or cautious) manner.	Careful handling involves workers showing attention to potential danger, error or harm and carrying out the activity in a very exact and thorough (or cautious) manner.	na	na	na	na	na	na
**Drop height**	Drop height < 0.5 m	na	na	na	na	na	na	na
**Containment level**	Open process	na	na	na	na	na	na	na
**OC: Surface contamination**								
	**WCS1**	**WCS2**	**WCS3**	**WCS4**	**WCS5**	**WCS6**	**WCS7**	**WCS8**
**Process fully enclosed?**	No	No	No	No	No	No	No	No
**Effective housekeeping practices in place?**	Yes	Yes	Yes	Yes	Yes	Yes	Yes	Yes
**Work area**	Indoors	Indoors	Indoors	Indoors	Indoors	Indoors	Indoors	Indoors
**Room size (m^3)**	3000	3000	3000	3000	3000	3000	3000	3000
**RMMs: Localised controls**								
	**WCS1**	**WCS2**	**WCS3**	**WCS4**	**WCS5**	**WCS6**	**WCS7**	**WCS8**
**Primary**	On-tool extraction (90.00 % reduction)	On-tool extraction (90.00 % reduction)	On-tool extraction (90.00 % reduction)	On-tool extraction (90.00 % reduction)	On-tool extraction (90.00 % reduction)	High level containment (99.90 % reduction)	On-tool extraction (90.00 % reduction)	On-tool extraction (90.00 % reduction)
**Secondary**	No localized controls	No localized controls	No localized controls	Medium level containment	No localized controls	No localized controls	Medium level containment	No localized controls

	(0.00 % reduction)	(0.00 % reduction)	(0.00 % reduction)	(99.00 % reduction)	(0.00 % reduction)	(0.00 % reduction)	(99.00 % reduction)	(0.00 % reduction)
**RMM: Dispersion**								
	**WCS1**	**WCS2**	**WCS3**	**WCS4**	**WCS5**	**WCS6**	**WCS7**	**WCS8**
**Segregartion**	na	na	na	na	na	na	No segregation (0.00 % reduction)	na
**Personal enclosure**	na	na	na	na	na	na	No personal enclosure (0.00 % reduction)	na
**Ventilation rate**	3 air changes per hour (ACH)	3 air changes per hour (ACH)	3 air changes per hour (ACH)	3 air changes per hour (ACH)	3 air changes per hour (ACH)	3 air changes per hour (ACH)	3 air changes per hour (ACH)	3 air changes per hour (ACH)

Table S5b - Company B - ART parameters. NF: Near-field exposure; FF: Far-field exposure; na: not available

	**WCS1**	**WCS2**	**WCS3**	**WCS4**	**WCS5**	**WCS6**	**WCS7**	**WCS8**
**Total duration (mins)**	10	10	10	10	300	20	20	60
**Non exposure period (mins)**	470	470	470	470	180	460	460	420
**Field exposure**	NF	NF	NF	NF	FF	NF	NF	NF
**OC: substance emission potential**								
	**WCS1**	**WCS2**	**WCS3**	**WCS4**	**WCS5**	**WCS6**	**WCS7**	**WCS8**
**Substance product type**	Powders, granules or pelletised material	Powders, granules or pelletised material	Powders dissolved in a liquid or incorporated in a liquid matrix	Powders dissolved in a liquid or incorporated in a liquid matrix	Powders dissolved in a liquid or incorporated in a liquid matrix	Powders dissolved in a liquid or incorporated in a liquid matrix	Powders dissolved in a liquid or incorporated in a liquid matrix	Powders dissolved in a liquid or incorporated in a liquid matrix
**Liquid matrix weight fraction**	na	na	Substantial	Substantial	Substantial	Substantial	Substantial	Substantial
**Viscosity**	na	na	Low	Low	Low	Low	Low	Medium
**Dustiness**	Granules, flakes or pellets	Granules, flakes or pellets	na	na	na	na	na	na

**Moisture content**	Dry product (< 5 % moisture content)	Dry product (< 5 % moisture content)	na	na	na	na	na	na
**Powder weight fraction**	Pure material	Pure material	na	na	na	na	na	na
**OC: Activity emission potential**								
	**WCS1**	**WCS2**	**WCS3**	**WCS4**	**WCS5**	**WCS6**	**WCS7**	**WCS8**
**Activity class**	Falling powders	Handling of contaminated solid objects or paste	Activities with relatively undisturbed surfaces (no aerosol formation)	Activities with relatively undisturbed surfaces (no aerosol formation)	Activities with agitated surfaces	Activities with agitated surfaces	Activities with agitated surfaces	Activities with relatively undisturbed surfaces (no aerosol formation)
**Situation**	Transferring 10 – 100 kg/minute	Handling of objects with limited residual dust (thin layer visible)	Open surface 1 - 3 m²	Open surface 1 - 3 m²	Open surface 1 - 3 m²	Open surface 1 - 3 m²	Open surface 1 - 3 m²	Open surface 1 - 3 m²
**Handling type**	Careful handling involves workers showing attention to potential danger, error or harm and carrying out the activity in a very exact and thorough (or cautious) manner	Careful handling involves workers showing attention to potential danger, error or harm and carrying out the activity in a very exact and thorough (or cautious) manner	na	na	na	na	na	na
**Drop height**	Drop height < 0.5 m	na	na	na	na	na	na	na
**Containment level**	Open process	na	na	na	na	na	na	na
**OC: Surface contamination**								
	**WCS1**	**WCS2**	**WCS3**	**WCS4**	**WCS5**	**WCS6**	**WCS7**	**WCS8**
**Process fully enclosed?**	No	No	No	No	No	No	No	No

**Effective housekeeping practices in place?**	Yes	Yes	Yes	Yes	Yes	Yes	Yes	Yes
**Work area**	Indoors	Indoors	Indoors	Indoors	Indoors	Indoors	Indoors	Indoors
**Room size (m³)**	1000	1000	1000	1000	1000	1000	1000	1000
**RMMs: Localised controls**								
	**WCS1**	**WCS2**	**WCS3**	**WCS4**	**WCS5**	**WCS6**	**WCS7**	**WCS8**
**Primary**	Fixed capturing hood (90.00 % reduction)	Fixed capturing hood (90.00 % reduction)	On-tool extraction (90.00 % reduction)	On-tool extraction (90.00 % reduction)	On-tool extraction (90.00 % reduction)	On-tool extraction (90.00 % reduction)	On-tool extraction (90.00 % reduction)	On-tool extraction (90.00 % reduction)
**Secondary**	No localized controls (0.00 % reduction)	No localized controls (0.00 % reduction)	Low level containment (90.00 % reduction)	Low level containment (90.00 % reduction)	Low level containment (90.00 % reduction)	Low level containment (90.00 % reduction)	Low level containment (90.00 % reduction)	Low level containment (90.00 % reduction)
**RMM: Dispersion**								
	**WCS1**	**WCS2**	**WCS3**	**WCS4**	**WCS5**	**WCS6**	**WCS7**	**WCS8**
**Segregartion**	na	na	na	na	No segregation (0.00 % reduction)	na	na	na
**Personal enclosure**	na	na	na	na	No personal enclosure (0.00 % reduction)	na	No personal enclosure (0.00 % reduction)	na
**Ventilation rate**	3 air changes per hour (ACH)	3 air changes per hour (ACH)	3 air changes per hour (ACH)	3 air changes per hour (ACH)	3 air changes per hour (ACH)	3 air changes per hour (ACH)	3 air changes per hour (ACH)	3 air changes per hour (ACH)

Table S5c - Company C - ART parameters. NF: Near-field exposure; FF: Far-field exposure; na: not available.

	WCS1	WCS2	WCS3	WCS4	WCS5	WCS6	WCS7	WCS8
**Total duration (mins)**	15	20	30	30	75	70	50	15
**Non exposure period (mins)**	465	460	450	450	405	410	430	465
**Field exposure**	NF	NF	NF	NF	NF	NF	FF	NF
**OC: substance emission potential**								
	**WCS1**	**WCS2**	**WCS3**	**WCS4**	**WCS5**	**WCS6**	**WCS7**	**WCS8**
**Substance product type**	Powders dissolved in a liquid or incorporated in a liquid matrix	Powders dissolved in a liquid or incorporated in a liquid matrix	Powders dissolved in a liquid or incorporated in a liquid matrix	Powders dissolved in a liquid or incorporated in a liquid matrix	Powders dissolved in a liquid or incorporated in a liquid matrix	Powders dissolved in a liquid or incorporated in a liquid matrix	Powders dissolved in a liquid or incorporated in a liquid matrix	Powders dissolved in a liquid or incorporated in a liquid matrix
**Liquid matrix weight fraction**	Main component	Main component	Main component	Minor	Main component	Main component	Small	Main component
**Viscosity**	Low	Low	Low	Low	Medium	Low	Low	Low
**OC: Activity emission potential**								
	**WCS1**	**WCS2**	**WCS3**	**WCS4**	**WCS5**	**WCS6**	**WCS7**	**WCS8**
**Activity class**	Bottom loading	Activities with relatively undisturbed surfaces (no aerosol formation)	Handling of contaminated objects	Handling of contaminated objects	Activities with relatively undisturbed surfaces (no aerosol formation)	Activities with agitated surfaces	Activities with relatively undisturbed surfaces (no aerosol formation)	Handling of contaminated objects
**Situation**	Transfer of liquid product with flow of 10 - 100 l/minute	Open surface 1 - 3 m²	Activities with treated/contaminated objects (surface 0.1-0.3 m²)	Activities with treated/contaminated objects (surface 0.1-0.3 m²)	Open surface 1 - 3 m²	Open surface 1 - 3 m²	Open surface 1 - 3 m²	Open surface > 3 m²
**Containment level**	na	na	Contamination < 10 % surface	Contamination < 10 % surface	na	na	na	Contamination < 10 % surface
**OC: Surface contamination**								
	**WCS1**	**WCS2**	**WCS3**	**WCS4**	**WCS5**	**WCS6**	**WCS7**	**WCS8**

**Process fully enclosed?**	No	No	No	No	No	No	No	No
**Effective housekeeping practices in place?**	Yes	Yes	Yes	Yes	Yes	No	Yes	Yes
**Work area**	Indoors	Indoors	Indoors	Indoors	Indoors	Indoors	Indoors	Indoors
**Room size (m³)**	3000	3000	3000	3000	3000	3000	3000	3000
**RMMs: Localised controls**								
	**WCS1**	**WCS2**	**WCS3**	**WCS4**	**WCS5**	**WCS6**	**WCS7**	**WCS8**
**Primary**	On-tool extraction (90.00 % reduction)	On-tool extraction (90.00 % reduction)	On-tool extraction (90.00 % reduction)	No localized controls	On-tool extraction (90.00 % reduction)	On-tool extraction (90.00 % reduction)	No localized controls	No localized controls
**Secondary**	Low level containment	Low level containment	Low level containment (90.00 % reduction)	No localized controls	No localized controls	High level containment (99.90 % reduction)	No localized controls (0.00 % reduction)	No localized controls (0.00 % reduction)
**RMM: Dispersion**								
	**WCS1**	**WCS2**	**WCS3**	**WCS4**	**WCS5**	**WCS6**	**WCS7**	**WCS8**
**Segregartion**	na	na	na	na	na	na	No segregation (0.00 % reduction)	na
**Personal enclosure**	na	na	na	na	na	na	No personal enclosure (0.00 % reduction)	na
**Ventilation rate**	10 air changes per hour (ACH)	10 air changes per hour (ACH)	10 air changes per hour (ACH)	10 air changes per hour (ACH)	10 air changes per hour (ACH)	10 air changes per hour (ACH)	Only good natural ventilation	1 air changes per hour (ACH)

Table S5d - Company D - ART parameters. NF: Near-field exposure; FF: Far-field exposure; na: not available.

	WCS2	WCS3	WCS4	WCS5	WCS6	WCS7	WCS8	WCS9	WCS10	WCS11	WCS12
**Total duration (mins)**	10	15	5	5	5	30	300	300	60	10	30
**Non exposure period (mins)**	470	465	475	475	475	450	180	180	420	470	450
**Field exposure**	NF	NF	NF	NF	NF	NF	FF	FF	NF	NF	NF
**OC: substance emission potential**											
	**WCS2**	**WCS3**	**WCS4**	**WCS5**	**WCS6**	**WCS7**	**WCS8**	**WCS9**	**WCS10**	**WCS11**	**WCS12**
**Substance product type**	Powders dissolved in a liquid or incorporated in a liquid matrix	Powders, granules or pelletised material	Powders, granules or pelletised material	Powders dissolved in a liquid or incorporated in a liquid matrix	Powders dissolved in a liquid or incorporated in a liquid matrix	Powders dissolved in a liquid or incorporated in a liquid matrix	Powders dissolved in a liquid or incorporated in a liquid matrix	Powders dissolved in a liquid or incorporated in a liquid matrix	Powders dissolved in a liquid or incorporated in a liquid matrix	Powders dissolved in a liquid or incorporated in a liquid matrix	Powders dissolved in a liquid or incorporated in a liquid matrix
**Liquid matrix weight fraction**	Main component	na	na	Substantial	Substantial	Very small	Substantial	Substantial	Very small	Very small	Substantial
**Viscosity**	Low	na	Na	Low	Low	Low	Low	Low	Low	Low	Low
**Dustiness**	na	Granules, flakes or pellets	Granules, flakes or pellets	na	na	na	na	na	na	na	na
**Moisture content**	na	Dry product (< 5 % moisture content)	Dry product (< 5 % moisture content)	na	na	na	na	na	na	na	na

**Powder weight fraction**	na	Pure material	Pure material	na	na	na	na	na	na	na	na
**OC: Activity emission potential**											
	**WCS2**	**WCS3**	**WCS4**	**WCS5**	**WCS6**	**WCS7**	**WCS8**	**WCS9**	**WCS10**	**WCS11**	**WCS12**
**Activity class**	Bottom loading	Bottom loading	Falling powders	Handling of contaminated objects or paste	Activities with relatively undisturbed surfaces (no aerosol formation)	Activities with relatively undisturbed surfaces (no aerosol formation)	Handling of contaminated objects	Activities with agitated surfaces	Activities with agitated surfaces	Handling of contaminated objects	Activities with relatively undisturbed surfaces (no aerosol formation)
**Situation**	Transfer of liquid product with flow of 10 - 100 l/minute	Transfer of liquid product with flow of 10 - 100 l/minute	Transferring 10 – 100 kg/minute	Handling of objects with limited residual dust (thin layer visible)	Open surface > 3 m²	Open surface > 3 m²	Activities with treated/contaminated objects (surface > 3 m²)	Open surface > 3 m²	Open surface > 3 m²	Activities with treated/contaminated objects (surface > 3 m²)	Open surface 1 - 3 m²
**Handling type**	na	na	Careful transfer involves workers showing attention to potential danger, error or harm and carrying out the activity in a very exact and thorough	Careful transfer involves workers showing attention to potential danger, error or harm and carrying out the activity in a very exact and thorough (or	na	na	na	na	na	na	na

			(or cautious) manner	cautious) manner							
**Drop height**	na	na	Drop height < 0.5 m	na	na	na	na	na	na	na	na
**Containment level**	na	na	Open process	na	na	na	Contamination < 10 % surface	na	na	Contamination < 10 % surface	na
**OC: Surface contamination**											
	**WCS2**	**WCS3**	**WCS4**	**WCS5**	**WCS6**	**WCS7**	**WCS8**	**WCS9**	**WCS10**	**WCS11**	**WCS12**
**Process fully enclosed?**	No	No	No	No	No	No	No	No	No	No	No
**Effective housekeeping practices in place?**	Yes	Yes	Yes	Yes	Yes	Yes	Yes	Yes	Yes	Yes	Yes
**Work area**	Indoors	Indoors	Indoors	Indoors	Indoors	Indoors	Indoors	Indoors	Indoors	Indoors	Indoors
**Room size (m^3)**	3000	3000	3000	3000	3000	3000	3000	3000	3000	100	3000
**RMMs: Localised controls**											
	**WCS2**	**WCS3**	**WCS4**	**WCS5**	**WCS6**	**WCS7**	**WCS8**	**WCS9**	**WCS10**	**WCS11**	**WCS12**
**Primary**	On-tool extraction (90.00 % reduction)	On-tool extraction (90.00 % reduction)	On-tool extraction (90.00 % reduction)	On-tool extraction (90.00 % reduction)	On-tool extraction (90.00 % reduction)	Fixed capturing hood (90.00 % reduction)	On-tool extraction (90.00 % reduction)	On-tool extraction (90.00 % reduction)	Fixed capturing hood (90.00 % reduction)	High level containment (99.90 % reduction)	On-tool extraction (90.00 % reduction)
**Secondary**	Other receiving hoods	Other receiving hoods	Other receiving hoods	Other receiving hoods	Other receiving hoods	Other receiving hoods	Other receiving hoods	Other receiving hoods (80.00 % reduction)	Other receiving hoods (80.00 % reduction)	No localized controls (0.00 % reduction)	Other receiving hoods (80.00 % reduction)
**RMM: Dispersion**											

	WCS2	WCS3	WCS4	WCS5	WCS6	WCS7	WCS8	WCS9	WCS10	WCS11	WCS12
**Segregartion**	na	na	na	na	na	na	No segregation (0.00 % reduction)	No segregation (0.00 % reduction	na	na	na
**Personal enclosure**	na	na	na	na	na	na	No personal enclosure (0.00 % reduction)	No personal enclosure (0.00 % reduction)	na	na	na
**Ventilation rate**	10 air changes per hour (ACH)	10 air changes per hour (ACH)	10 air changes per hour (ACH)	10 air changes per hour (ACH)	10 air changes per hour (ACH)	10 air changes per hour (ACH)	10 air changes per hour (ACH)	10 air changes per hour (ACH)	10 air changes per hour (ACH)	1 air changes per hour (ACH)	10 air changes per hour (ACH)

SECTION C - Comparison between outcomes from ART web and TREXMO

Table S6a - Company A - Comparison between different ART tools (i.e. ART web and TREXMO 2.0).

	ART web		Trexmo +		Trexmo	
**WCS**	**P90 (mg/m³) full-shift exposure**	**CI 90% (mg/m³)**	**P90 (CI 90%) (mg/ m³) Full-shift exposure**	**CI 90% (mg/ m³)**	**P90 (CI 90%) (mg/ m³) Full-shift exposure**	**CI 90% (mg/ m³)**
1	1.60E-03	7.90E-04-3.30E-03	1.60E-03	3.00E-04-1.20E-02	1.60E-03	2.90E-04-1.30E-02
2	1.00E-04	5.20E-05-2.20E-04	1.10E-04	2.00E-05-8.10E-04	1.00E-05	2.00E-06-7.90E-05
3	3.20E-06	1.40E-06-7.50E-06	3.20E-06	6.00E-07-2.55E-05	3.25E-06	4.80E-07-3.15E-05
4	1.60E-05	7.30E-06-3.80E-05	1.61E-05	3.05E-06-1.31E-04	1.63E-05	3.35E-06-6.60E-05
5	5.80E-05	2.60E-05-1.30E-04	5.65E-05	1.09E-05-4.40E-04	5.70E-05	8.90E-06-5.45E-04
6	2.00E-09	9.00E-10-4.70E-09	2.00E-09	2.56E-08-1.50E-08	2.05E-09	2.95E-10-1.85E-08
7	2.30E-05	1.00E-05-5.30E-05	2.22E-05	4.20E-06-1.68E-04	2.17E-05	3.30E-06-2.14E-04
8	3.20E-06	1.40E-06-7.40E-06	3.20E-06	6.50E-07-2.35E-05	3.20E-06	9.50E-07-2.61E-05

Table S6b - Company B - Comparison between different ART tools (i.e. ART web and TREXMO 2.0).

	ART web		Trexmo +		Trexmo	
**WCS**	**P90 (mg/m³) full-shift exposure**	**CI 90% (mg/m³)**	**P90 (CI 90%) (mg/m3) Full-shift exposure**	**CI 90% (mg/m3)**	**P90 (CI 90%) (mg/m3) Full-shift exposure**	**CI 90% (mg/m³)**
1	3.60E-03	1.80E-03-7.50E-03	3.50E-03	6.80E-04-2.60E-02	3.60E-03	7.00E-04-2.70E-02
2	1.20E-04	5.90E-05-2.50E-04	1.20E-04	2.40E-05-9.40E-04	1.20E-04	2.30E-05-8.60E-04
3	1.80E-06	8.20E-07-4.30E-06	1.80E-06	3.45E-07-1.34E-05	1.86E-06	2.70E-07-1.69E-05
4	1.80E-06	8.30E-07-4.30E-06	1.81E-06	3.60E-07-1.41E-05	1.75E-06	2.71E-07-1.74E-05
5	7.00E-04	3.10E-04-1.60E-03	7.15E-04	1.37E-04-5.40E-03	7.15E-04	1.02E-04-6.65E-03
6	3.70E-04	1.70E-04-8.50E-04	3.65E-04	7.15E-05-2.83E-03	3.70E-04	5.40E-05-3.50E-03
7	3.70E-04	1.70E-04-8.50E-04	3.65E-04	7.10E-05-2.78E-03	3.70E-04	5.40E-05-3.60E-03
8	3.30E-06	1.50E-06-7.70E-06	3.25E-06	6.55E-07-2.41E-05	3.25E-06	2.78E-06-3.25E-05

Table S6c -Company C - Comparison between different ART tools (i.e. ART web and TREXMO 2.0).

	ART web		Trexmo +		Trexmo	
**WCS**	**P90 (mg/m³) full-shift exposure**	**CI 90% (mg/m³)**	**P90 (CI 90%) (mg/ m³) Full-shift exposure**	**CI 90% (mg/ m³)**	**P90 (CI 90%) (mg/ m³) Full-shift exposure**	**CI 90% (mg/m³)**
1	5.60E-06	2.50E-06-1.30E-05	5.55E-06	1.08E-06-4.15E-05	5.60E-06	8.50E-07-5.50E-05
2	7.50E-06	3.40E-06-1.70E-05	7.40E-06	1.39E-06-5.85E-05	7.40E-06	1.10E-06-6.95E-05
3	1.10E-06	5.10E-07-2.60E-06	1.10E-06	2.25E-07-8.50E-06	1.10E-06	1.65E-07-1.09E-05
4	1.20E-05	5.40E-06-2.80E-05	1.21E-05	2.30E-06-9.00E-05	1.20E-07	1.80E-08-1.13E-06
5	8.50E-05	3.80E-05-2.00E-04	8.00E-05	1.60E-05-6.40E-04	8.50E-05	1.27E-05-7.50E-04
6	2.60E-05	1.20E-05-6.10E-05	2.65E-05	1.28E-05-1.95E-04	2.65E-05	3.80E-06-2.45E-04
7	5.60E-06	2.50E-06-1.30E-05	5.45E-06	1.08E-06-4.25E-05	5.40E-06	7.85E-07-5.15E-05
8	6.50E-06	2.90E-06-1.50E-05	6.40E-06	1.23E-06-4.75E-05	6.50E-06	9.40E-07-6.00E-05

Table S6d - Company D - Comparison between different ART tools (i.e. ART web and TREXMO 2.0).

	ART web		Trexmo +		Trexmo		
**WCS**	**P90 (mg/m³) full-shift exposure**	**CI 90% (mg/m³)**	**P90 (mg/m³) Full-shift exposure**	**P90 (CI 90%) (mg/ m³) Full-shift exposure**	**CI 90% (mg/ m³)**	**P90 (CI 90%) (mg/ m³) Full-shift exposure**	**CI 90% (mg/m³)**
2	7.50E-06	3.40E-06-1.80E-05	7.50E-06	7.40E-06	1.40E-06-5.85E-05	7.40E-06	1.09E-06-7.05E-05
3	9.40E-04	4.70E-04-2.00E-03	9.38E-04	9.80E-04	1.90E-04-7.70E-03	9.60E-04	1.90E-04-7.40E-03
4	1.10E-05	5.20E-06-2.20E-05	1.00E-03	1.10E-05	2.00E-06-8.40E-05	1.10E-05	2.10E-06-8.90E-05
5	1.60E-06	7.20E-07-3.70E-06	1.67E-06	1.57E-06	5.25E-07-1.11E-05	1.62E-06	2.39E-07-1.56E-05
6	1.60E-06	7.30E-07-3.70E-06	1.56E-06	1.57E-06	5.35E-07-1.01E-05	1.62E-06	2.85E-06-1.57E-05
7	2.40E-08	1.10E-08-5.50E-08	2.44E-08	2.35E-08	4.65E-09-1.85E-07	2.40E-08	3.50E-09-2.30E-07
8	4.40E-04	2.00E-04-1.00E-03	4.44E-04	4.10E-04	7.80E-05-3.05E-03	4.15E-04	6.00E-05-3.90E-03
9	4.40E-04	2.00E-04-1.00E-03	7.10E-04	4.15E-04	7.80E-05-3.25E-03	4.10E-04	6.00E-05-3.80E-03

10	4.90E-08	2.20E-08-1.10E-07	4.88E-08	4.85E-08	9.65E-09-3.70E-07	4.80E-08	6.90E-09-4.50E-07
11	1.00E-08	4.60E-09-2.40E-08	4.70E-07	1.05E-08	2.00E-09-7.75E-08	2.10E-09	3.00E-10-1.95E-08
12	2.90E-06	1.30E-06-6.70E-06	3.00E-06	2.89E-06	5.55E-07-2.17E-05	2.93E-06	4.35E-07-2.47E-05

Comparison between outcomes from measured data (personal and stationary) and estimate data from ART Web

Table S7a - Company A – Comparison between estimated data (ART web). ***AM: arithmetic mean, MAX: maximum.***

WCS	Type of data	Estimate data	Measured data (mg/m^3)			Estimate factor		
			AM	MAX	90th perc	AM	MAX	90th perc
1	personal	1.60E-03	5.00E-05	5.00E-05	5.00E-05	32.00	32.00	32.00
2	personal	1.00E-04	5.00E-05	5.00E-05	5.00E-05	2.00	2.00	2.00
3	personal	3.20E-06	5.00E-05	5.00E-05	5.00E-05	0.06	0.06	0.06
4	personal	1.60E-05	5.00E-05	5.00E-05	5.00E-05	0.32	0.32	0.32
5	personal	5.80E-05	5.00E-05	5.00E-05	5.00E-05	1.16	1.16	1.16
6	personal	2.00E-09	-	-	-	-	-	-
7	personal	2.30E-05	5.00E-05	5.00E-05	5.00E-05	0.46	0.46	0.46
8	personal	3.20E-06	5.00E-05	5.00E-05	5.00E-05	0.06	0.06	0.06
1	stationary	1.60E-03	8.00E-05	2.00E-04	1.82E-04	20.00	8.00	8.79
2	stationary	1.00E-04	8.00E-05	2.00E-04	1.82E-04	1.25	0.50	0.55
3	stationary	3.20E-06	8.00E-05	2.00E-04	1.82E-04	0.04	0.02	0.02
4	stationary	1.60E-05	8.00E-05	2.00E-04	1.82E-04	0.20	0.08	0.09
5	stationary	5.80E-05	8.00E-05	2.00E-04	1.82E-04	0.73	0.29	0.32
6	stationary	2.00E-09	5.00E-05	5.00E-05	5.00E-05	0.00004	0.00004	0.00004
7	stationary	2.30E-05	1.10E-04	2.00E-04	2.00E-04	0.21	0.12	0.12
8	stationary	3.20E-06	8.00E-05	2.00E-04	1.82E-04	0.04	0.02	0.02

Table S7b - Company B – Comparison between estimate data (ART web). ***AM: arithmetic mean, MAX: maximum.***

WCS	Type of data	Estimate data	Measured data (mg/m^3)			Estimate factor		
			AM	MAX	90th perc	AM	MAX	90th perc
1	personal	3.60E-03	0.002	2.00E-03	1.76E-03	4.1656	1.8000	2.0455
2	personal	1.20E-04	0.002	2.00E-03	1.76E-03	0.1389	0.0600	0.0682
3	personal	1.80E-06	0.002	2.00E-03	1.76E-03	0.0021	0.0009	0.0010
4	personal	1.80E-06	0.002	2.00E-03	1.76E-03	0.0021	0.0009	0.0010
5	personal	7.00E-04	0.002	2.00E-03	1.76E-03	0.8100	0.3500	0.3977
6	personal	3.70E-04	0.002	2.00E-03	1.76E-03	0.4281	0.1850	0.2102
7	personal	3.70E-04	0.002	2.00E-03	1.76E-03	0.4281	0.1850	0.2102
8	personal	3.30E-06	0.002	2.00E-03	1.76E-03	0.0038	0.0017	0.0019
1	stationary	3.60E-03	9.59E-04	2.00E-03	1.79E-03	3.7529	1.8000	2.0112
2	stationary	1.20E-04	9.59E-04	2.00E-03	1.79E-03	0.1251	0.0600	0.0670
3	stationary	1.80E-06	9.59E-04	2.00E-03	1.79E-03	0.0019	0.0009	0.0010
4	stationary	1.80E-06	9.59E-04	2.00E-03	1.79E-03	0.0019	0.0009	0.0010
5	stationary	7.00E-04	9.59E-04	2.00E-03	1.79E-03	0.7297	0.3500	0.3911
6	stationary	3.70E-04	9.59E-04	2.00E-04	1.79E-03	0.3857	0.1850	0.2067
7	stationary	3.70E-04	8.00E-05	2.00E-04	1.79E-03	0.3857	0.1850	0.2067
8	stationary	3.30E-06	8.00E-05	2.00E-04	1.79E-03	0.0034	0.0017	0.0018

Table S7c - Company C – Comparison between estimated data (ART web). ***AM: arithmetic mean, MAX: maximum.***

WCS	Type of data	Estimate data	Measured data (mg/m^3)			Estimate factor		
			AM	MAX	90th perc	AM	MAX	90th perc
1	personal	5.60E-06	1.88E-03	3.80E-03	3.26E-03	0.0030	0.0015	0.0017
2	personal	7.50E-06	1.88E-03	3.80E-03	3.26E-03	0.0040	0.0020	0.0023
3	personal	1.10E-06	1.88E-03	3.80E-03	3.26E-03	0.0006	0.0003	0.0003
4	personal	1.20E-05	1.88E-03	3.80E-03	3.26E-03	0.0064	0.0032	0.0037
5	personal	8.50E-05	1.88E-03	3.80E-03	2.90E-03	0.0453	0.0224	0.0261
6	personal	2.60E-05	1.75E-03	3.80E-03	2.90E-03	0.0149	0.0068	0.0090
7	Personal	5.60E-06	2.50E-03	3.00E-03	2.90E-03	0.0022	0.0019	0.0019
8	personal	6.50E-06	2.50E-03	3.00E-03	2.90E-03	0.0026	0.0022	0.0022
1	stationary	5.60E-06	1.55E-03	4.00E-03	2.60E-03	0.0036	0.0014	0.0022
2	stationary	7.50E-06	1.55E-03	4.00E-03	2.60E-03	0.0048	0.0019	0.0029
3	stationary	1.10E-06	1.55E-03	4.00E-03	2.60E-03	0.0007	0.0003	0.0004
4	stationary	1.20E-05	1.55E-03	4.00E-03	2.60E-03	0.0077	0.0030	0.0046
5	stationary	8.50E-05	1.55E-03	4.00E-03	2.60E-03	0.0548	0.0213	0.0327
6	stationary	2.60E-05	1.55E-03	4.00E-03	2.60E-03	0.0168	0.0065	0.0100
7	stationary	5.60E-06	1.27E-03	3.00E-03	2.50E-03	0.0044	0.0019	0.0022
8	stationary	6.50E-06	1.27E-03	3.00E-03	2.50E-03	0.0051	0.0022	0.0026

Table S7d - Company D – Comparison between estimate data (ART web). ***AM: arithmetic mean, MAX: maximum.***

WCS	Type of data	Estimate data	Measured data (mg/m^3)			Estimate factor		
			AM	MAX	90th perc	AM	MAX	90th perc
2	personal	7.50E-06	3.03E-04	1.79E-03	5.60E-04	0.02	0.004	0.01
3	personal	9.40E-04	3.03E-04	1.79E-03	5.60E-04	3.10630	0.525140	1.67887
4	personal	1.10E-05	3.03E-04	1.79E-03	5.60E-04	0.03635	0.006145	0.01965
5	personal	1.60E-06	3.03E-04	1.79E-03	5.60E-04	0.00529	0.000894	0.00286
6	personal	1.60E-06	3.03E-04	1.79E-03	5.60E-04	0.00529	0.000894	0.00286
7	personal	2.40E-08	3.03E-04	1.79E-03	5.60E-04	0.00008	0.000013	0.00004
8	personal	4.40E-04	3.03E-04	1.79E-03	5.60E-04	1.45401	0.245810	0.78585
9	personal	4.40E-04	3.03E-04	1.79E-03	5.60E-04	1.45401	0.245810	0.78585
10	personal	4.90E-08	3.03E-04	1.79E-03	5.60E-04	0.00016	0.000027	0.00009
11	personal	1.00E-08	3.03E-04	1.79E-03	5.60E-04	0.00003	0.000006	0.00002
12	personal	2.90E-06	3.03E-04	1.79E-03	5.60E-04	0.00958	0.001620	0.00518
2	stationary	7.50E-06	9.00E-04	9.00E-03	2.16E-03	0.01	0.0008	0.0035
3	stationary	9.40E-04	9.00E-04	9.00E-03	2.16E-03	1.04485	0.104444	0.435185
4	stationary	1.10E-05	9.00E-04	9.00E-03	2.16E-03	0.01223	0.001222	0.005093
5	stationary	1.60E-06	9.00E-04	9.00E-03	2.16E-03	0.00178	0.000178	0.000741
6	stationary	1.60E-06	9.00E-04	9.00E-03	2.16E-03	0.00178	0.000178	0.000741
7	stationary	2.40E-08	9.00E-04	9.00E-03	2.16E-03	0.00003	0.000003	0.000011
8	stationary	4.40E-04	9.00E-04	9.00E-03	2.16E-03	0.48908	0.048889	0.203704
9	stationary	4.40E-04	9.00E-04	9.00E-03	2.16E-03	0.48908	0.048889	0.203704
10	stationary	4.90E-08	9.00E-04	9.00E-03	2.16E-03	0.00005	0.000005	0.000023
11	stationary	1.00E-08	9.00E-04	9.00E-03	2.16E-03	0.00001	0.000001	0.000005
12	stationary	2.90E-06	9.00E-04	9.00E-03	2.16E-03	0.00322	0.000322	0.001343

Table S8a - Company A. Level of conservatism *E: exposure estimates; M: measured data.*

	Personal					Stationary				
WCS	E>M (n)	%	E<M (n)	%	Level of conservatism	E>M (n)	%	E<M (n)	%	Level of conservatism
1	3/3	100	0/3	0	high	2/4	50	2/4	50	low
2	3/3	100	0/3	0	high	4/4	100	0/4	0	high
3	0/3	0	3/3	100	low	3/4	75	1/4	25	mean
4	0/3	0	3/3	100	low	0/4	0	4/4	100	low
5	3/3	100	0/3	0	high	0/4	0	4/4	100	low
6	-	-	-	-	-	3/4	75	1/4	25	mean
7	0/9	0	9/9	100	high	2/4	50	2/4	50	low
8	0/3	0	9/9	100	high	0/4	0	4/4	100	low

Table S8b - Company B. Level of conservatism. *E: exposure estimates; M: measured data.*

	Personal					Stationary				
WCS	N E>M	%	N E<M	%	Level of conservatism	N E>M	%	N E<M	%	Level of conservatism
1	9/9	100	0/9	0	high	9/9	100	0/9	0	high
2	2/9	22	7/9	78	low	2/9	22	7/9	78	low
3	0/9	0	9/9	100	low	0/9	0	9/9	100	low
4	0/9	0	9/9	100	low	0/9	0	9/9	100	low
5	3/9	33	6/9	66	low	3/9	33	6/9	67	low
6	1/9	11	8/9	89	low	3/9	33	6/9	67	low
7	2/9	22	7/9	78	low	3/9	33	6/9	67	low
8	0/9	0	9/9	100	low	3/9	33	6/9	67	low

Table S8c - Company C. Level of conservatism. ***E: exposure estimates; M: measured data.***

	Personal					Stationary				
**WCS**	**N E>M**	**%**	**N E<M**	**%**	**Level of conservatism**	**N E>M**	**%**	**N E<M**	**%**	**Level of conservatism**
1	0/4	0	4/4	100	low	0/8	0	8/8	100	low
2	0/4	0	4/4	100	low	0/8	0	8/8	100	low
3	0/5	0	5/5	100	low	0/7	0	7/7	100	low
4	0/5	0	5/5	100	low	0/9	0	9/9	100	low
5	0/5	0	5/5	100	low	0/8	0	8/8	100	low
6	0/6	0	6/6	100	low	0/8	0	8/8	100	low
7	0/2	0	2/2	100	low	0/6	0	6/6	100	low
8	0/2	0	2/2	100	low	0/6	0	6/6	100	low

Table S8d - Company D. Level of conservatism. ***E: exposure estimates; M: measured data.***

	Personal					Stationary				
**WCS**	**N E>M**	**%**	**N E<M**	**%**	**Level of conservatism**	**N E>M**	**%**	**N E<M**	**%**	**Level of conservatism**
2	0/18	0	18/18	100	low	0/20	0	20/20	100	low
3	16/18	89	2/18	11	high	16/20	80	4/20	20	mean
4	0/18	0	18/18	100	low	0/20	0	20/20	100	low
5	0/18	0	18/18	100	low	0/20	0	20/20	100	low
6	0/18	0	18/18	100	low	0/20	0	20/20	100	low
7	0/18	0	18/18	100	low	0/20	0	20/20	100	low
8	18/18	100	0/18	0	high	15/20	75	5/20	25	mean
9	18/18	100	0/18	0	high	15/20	75	5/20	25	mean
10	0/18	0	18/18	100	low	0/20	0	20/20	100	low
11	0/18	0	18/18	100	low	0/20	0	20/20	100	low
12	0/18	0	18/18	100	low	0/20	0	20/20	100	low

Section D - Excess Risk under REACH and Risk Characterization Ratio under OSH

Table S9 - Excess Risk and RCR divided in personal and stationary data and estimate and measurements.

Company	WCS	Excess Risk with exposure estimates	RCRp	RCRe	RCRs
A	1	1.28E-04	0.01	0.32	0.022
A	2	8.00E-06	0.01	0.02	0.016
A	3	1.28E-06	0.01	0.00064	0.016
A	4	6.40E-06	0.01	0.0032	0.016
A	5	5.27E-08	0.01	0.0116	0.016
A	6	1.60E-10	-	0.0116	0.016
A	7	9.20E-05	0.01	0.0046	0.01
A	8	1.28E-06	0.01	0.00064	0.016
B	1	1.38E-04	0.17	0.72	0.17
B	2	4.61E-06	0.17	0.024	0.17
B	3	8.64E-09	0.17	0.00036	0.17
B	4	6.91E-08	0.17	0.00036	0.17
B	5	2.80E-03	0.17	0.14	0.17
B	6	7.40E-05	0.17	0.074	0.17
B	7	7.40E-05	0.17	0.074	0.17
B	8	5.28E-09	0.17	0.00066	0.17
C	1	9.57E-07	0.38	0.76	0.31
C	2	3.00E-06	0.38	0.76	0.31
C	3	4.40E-06	0.45	0.76	0.3
C	4	4.80E-05	0.45	0.76	0.32
C	5	3.40E-05	0.45	0.76	0.31
C	6	1.04E-05	0.35	0.76	0.31
C	7	2.24E-06	0.5	0.4	0.25
C	8	3.55E-06	0.5	0.4	0.25

D	2	2.40E-07	0.06	0.0015	0.18
D	3	6.02E-05	0.06	0.19	0.18
D	4	7.04E-07	0.06	0.0022	0.18
D	5	2.56E-09	0.06	0.00032	0.18
D	6	6.40E-07	0.06	0.00032	0.18
D	7	9.60E-08	0.06	0.0000048	0.18
D	8	1.76E-03	0.06	0.088	0.18
D	9	1.76E-03	0.06	0.088	0.18
D	10	1.96E-08	0.06	0.00001	0.18
D	11	4.00E-09	0.06	0.000002	0.18
D	12	4.64E-09	0.06	0.00058	0.18

Table S10 - Exposure modifying factor.

Company	WCS	Frequency (days/year)	workdays/year	Exposure modifying factor for frequency (EMF freq) (frequency/workdays)	Exposure modifying factor for RPE (EMF RPE)	Total modifying factor (EMF freq/EMF RPE)
A	1	88	220	4.00E-01	5.00E-02	2.00E-02
A	2	88	220	4.00E-01	5.00E-02	2.00E-02
A	3	220	220	1.00E+00	1.00E-01	1.00E-01
A	4	220	220	1.00E+00	1.00E-01	1.00E-01
A	5	1	220	4.55E-03	5.00E-02	2.27E-04
A	6	44	220	2.00E-01	1.00E-01	2.00E-02
A	7	220	220	1.00E+00	1.00E+00	1.00E+00
A	8	220	220	1.00E+00	1.00E-01	1.00E-01
B	1	48	250	1.92E-01	5.00E-02	9.60E-03
B	2	48	250	1.92E-01	5.00E-02	9.60E-03
B	3	6	250	2.40E-02	5.00E-02	1.20E-03

B	4	48	250	1.92E-01	5.00E-02	9.60E-03
B	5	250	250	1.00E+00	1.00E+00	1.00E+00
B	6	250	250	1.00E+00	5.00E-02	5.00E-02
B	7	250	250	1.00E+00	5.00E-02	5.00E-02
B	8	2	250	8.00E-03	5.00E-02	4.00E-04
C	1	94	220	4.27E-01	1.00E-01	4.27E-02
C	2	220	220	1.00E+00	1.00E-01	1.00E-01
C	3	220	220	1.00E+00	1.00E+00	1.00E+00
C	4	220	220	1.00E+00	1.00E+00	1.00E+00
C	5	220	220	1.00E+00	1.00E-01	1.00E-01
C	6	220	220	1.00E+00	1.00E-01	1.00E-01
C	7	220	220	1.00E+00	1.00E-01	1.00E-01
C	8	30	220	1.36E-01	1.00E+00	1.36E-01
D	2	20	250	8.00E-02	1.00E-01	8.00E-03
D	3	40	250	1.60E-01	1.00E-01	1.60E-02
D	4	40	250	1.60E-01	1.00E-01	1.60E-02
D	5	1	250	4.00E-03	1.00E-01	4.00E-04
D	6	250	250	1.00E+00	1.00E-01	1.00E-01
D	7	250	250	1.00E+00	1.00E+00	1.00E+00
D	8	250	250	1.00E+00	1.00E+00	1.00E+00
D	9	250	250	1.00E+00	1.00E+00	1.00E+00
D	10	250	250	1.00E+00	1.00E-01	1.00E-01
D	11	250	250	1.00E+00	1.00E-01	1.00E-01
D	12	2	250	8.00E-03	5.00E-02	4.00E-04
